# Assessment of Nonfatal Bleeding Events as a Surrogate for Mortality in Coronary Artery Disease

**DOI:** 10.1016/j.jacadv.2023.100276

**Published:** 2023-02-20

**Authors:** Toshiki Kuno, Atsuyuki Watanabe, Yoshihisa Miyamoto, Leandro Slipczuk, Shun Kohsaka, Deepak L. Bhatt

**Affiliations:** a Division of Cardiology, Montefiore Medical Center, Albert Einstein College of Medicine, New York, New York, USA; b Department of Medicine, University of Tsukuba, Tsukuba, Japan; c National Cancer Center Institute for Cancer Control, Tokyo, Japan; d Division of Nephrology and Endocrinology, The University of Tokyo Hospital, Tokyo, Japan; e Department of Cardiology, Keio University School of Medicine, Tokyo, Japan; f Mount Sinai Heart, Icahn School of Medicine at Mount Sinai Health System, New York, New York, USA

**Keywords:** bleeding, coronary artery disease, meta-analysis, mortality, surrogate

## Abstract

**Background:**

Bleeding events are frequently applied as safety end points for randomized controlled trials (RCTs) investigating the effect of antithrombotic agents in patients with coronary artery disease. However, whether a bleeding event is a valid surrogate for death remain uncertain.

**Objectives:**

This study aimed to assess the correlation between the treatment effect on bleeding events and mortality.

**Methods:**

Multiple databases were searched to identify RCTs studying antithrombotic agents for patients with coronary artery disease through August 2022. Major and minor bleeding events were defined in included trials, mostly defined with BARC (Bleeding Academic Research Consortium) or TIMI (Thrombolysis In Myocardial Infarction) criteria. Trial-level correlations between nonfatal bleeding events and mortality were assessed. We performed subgroup analyses by the definitions of bleeding (BARC vs TIMI criteria), study year, and follow-up duration. We used a cutoff with a lower limit of 95% confidence interval of R^2^ >0.72 as a strong correlation and with an upper limit of 95% confidence interval of R^2^ <0.50 as a weak correlation.

**Results:**

A total of 48 RCTs with 181,951 participants were analyzed. Overall, trial-level R^2^ for major and minor bleeding were 0.09 (95% CI: 0.00-0.26) and 0.09 (95% CI: 0.00-0.27) for all-cause or cardiovascular death, respectively. When confined to major bleeding, R^2^ were 0.03 (95% CI: 0.00-0.13) and 0.01 (95% CI: 0.00-0.05), respectively. All of the subgroup analyses did not show any significant correlations.

**Conclusions:**

We demonstrated a trial-defined bleeding event may not be a valid surrogate for mortality in RCTs investigating the effect of antithrombotic agents for coronary artery disease.

A bleeding event is considered as a primary safety outcome in randomized controlled trials (RCTs) investigating antithrombotic agents for patients with coronary artery disease (CAD). The significance of bleeding events has increased over the last decade, accompanied by the advancement of drug-eluting stent or percutaneous coronary intervention techniques including intravascular imaging.^1^^,^^2^ Furthermore, various regimens for antithrombotic agents for CAD have been tested to decrease the bleeding event rates.^3^, ^4^, ^5^, ^6^, ^7^, ^8^, ^9^, ^10^, ^11^, ^12^, ^13^, ^14^, ^15^, ^16^ In general, it is assumed that bleeding events lead to worse long-term prognosis, including increased risk of death.^17^ In addition, a composite of major adverse cardiovascular events and bleeding outcomes as net adverse clinical events (NACE) has been used as the primary end point in RCTs, though not every trialist agrees with combining ischemic and bleeding end points.^18^, ^19^, ^20^, ^21^

Surrogate end points, including bleeding events, are used frequently in RCTs since they usually occur earlier than death enabling RCTs to be conducted with shorter follow-up periods, smaller sample sizes, and lower costs, which allows patients to access new therapies earlier.^22^ However, it remains uncertain whether a bleeding event can be a strict surrogate of death as part of a composite outcome.^14^^,^^23^ Indeed, a previous study demonstrated that bleeding events could lead to bleeding-related deaths, especially in the early phase after acute coronary syndrome or percutaneous coronary intervention, but not lead to non-bleeding-related death.^17^^,^^24^ Moreover, a recent study revealed that even myocardial infarction is not a surrogate of death in trials to treat or prevent CAD.^25^ The association between the treatment effect on a bleeding end point as a surrogate end point and the treatment effect on mortality as a final end point in a meta-analysis of RCTs had not been performed.^17^

Herein, our aim of this study was to assess the correlation between the treatment effect of an intervention such as shortened dual antiplatelet therapy (DAPT) duration on bleeding events and the treatment effect of the same intervention on all-cause or cardiovascular mortality. To clarify this correlation, we performed a meta-analysis of RCTs which investigated bleeding events and mortality in RCTs investigating the effect of antithrombotic agents for patients with CAD.

## Methods

This analysis was conducted according to the Preferred Reporting Items for Systematic Reviews and Meta-Analyses guidelines.^26^ The protocol was registered to the Prospective Register of Systematic Reviews (CRD 42022356035). Institutional review board/ethics exemption was granted for the design of the study.

### Eligibility criteria

The eligibility criteria were as follows: 1) the study was published in a peer-reviewed journal; 2) the design was a RCT with at least 2 different antithrombotic agents (DAPT strategies) for patients with CAD; 3) the study investigated mortality (all-cause and/or cardiovascular death) and a trial defined major or major/minor bleeding outcome as the safety endpoint; and 4) a sample size ≥500 patients.^27^

The trial defined major/minor bleedings were defined using the definition used in each trial as the safety end point; BARC (Bleeding Academic Research Consortium) type 2, 3, or 5 bleeding,^28^ BARC type 2, 3, 4, or 5 bleeding, or TIMI (Thrombolysis In Myocardial Infarction) major or minor bleeding was used.^29^ Similarly, the trial defined major bleeding was described with the definition which was used in each trial as the safety end point; BARC type 3 or 5 bleeding, BARC type 3, 4, or 5 bleeding,^28^ TIMI major bleeding, or GUSTO (Global Utilization of Streptokinase and Tissue Plasminogen Activator for Occluded Arteries) moderate or severe bleeding was used.^28^^,^^29^

### Information sources and data collection process

PubMed, EMBASE, and Cochrane CENTRAL databases were searched by a medical librarian with expertise in performing systematic reviews to identify all studies published on April 27, 2022, that investigated the effect of antithrombotic agents for patients with CAD. The extensive search strategy is shown in Supplemental Tables 1 to 3. Additional studies were added via a manual search of other sources, including references from identified articles, systematic reviews or meta-analyses, and commentaries. Two authors (A.W and T.K.) explored the search results to select studies according to the inclusion criteria. Then, these 2 authors reviewed the studies and independently judged the selection and outcomes with the Cochrane Collaboration risk of bias 2.0 tool.^30^

Extracted variables included study subjects, year of the first patient enrollment, the total number of randomized participants, numbers of participants by intervention or control groups, median follow-up duration, and definition of trial defined major or major/minor bleeding. Extracted outcomes included the number of all-cause and cardiovascular death, trial defined major or major/minor bleeding, and odds ratio (OR) or HR of treatment effect for all-cause and cardiovascular mortality, trial defined major or major/minor bleeding (if reported).

### Statistical analysis

We calculated odds ratio as a relative measure of effect from the number of events and the number of individuals in the intervention and control groups in each RCT. To confirm the correlation between all-cause or cardiovascular mortality and trial defined major/minor or major bleeding outcomes visually, we plotted the logarithm of odds ratios (log-ORs) for bleeding outcomes on the X coordinate and those for all-cause or cardiovascular mortality on the Y coordinate. We regressed log-ORs for all-cause or cardiovascular mortality on log-ORs for bleeding outcomes, weighted by the number of enrolled patients in each study, both of which were recommended for a trial-level correlation.^31^ We reported the slope, intercept, and the coefficient of determination (R^2^ of 1 indicates that the regression predictions fit perfectly) in these models. We repeated the analysis using the only articles which reported HR. We assessed the between-study heterogeneity by the I^2^ value (ranging from 0% to 100%).

To evaluate the trial-level association between the treatment effects on surrogate end point and mortality, the coefficient of determination (R^2^) was used. We estimated 95% confidence interval (CI) of R^2^ using a formula implemented in R package Surrogate.^32^^,^^33^ The R^2^ takes a value from 0 to 1 and the R^2^ 0 means the absence of surrogacy and 1 indicates perfect marker of surrogacy. We used a cut-off with a lower limit of 95% CI of R^2^ >0.72 as a strong correlation and with an upper limit of 95% CI of R^2^ <0.50 as a weak correlation.^34^

After conducting the analysis overall, we performed the following prespecified subgroup analysis: 1) bleeding outcomes defined only with BARC bleeding criteria; 2) bleeding outcomes defined only with TIMI bleeding criteria; 3) the first year of patient enrollment (the era of the trial, before 2010 vs after 2011) according to the previous article;^25^ 4) follow-up duration (>12 months vs ≤12 months); 5) trials in East Asia or non-East-Asia; 6) patients with acute coronary syndrome; and 7) different cutoff points of the following criteria; R^2^ ≥0.7 as strong, between 0.5 and 0.69 as moderate, and <0.5 as a weak correlation.^31^ We performed a meta-analysis of OR or HR extracted from enrolled article, using R software (R.4.1.2) with metafor and Surrogacy package.

## Results

**Figure 1:**
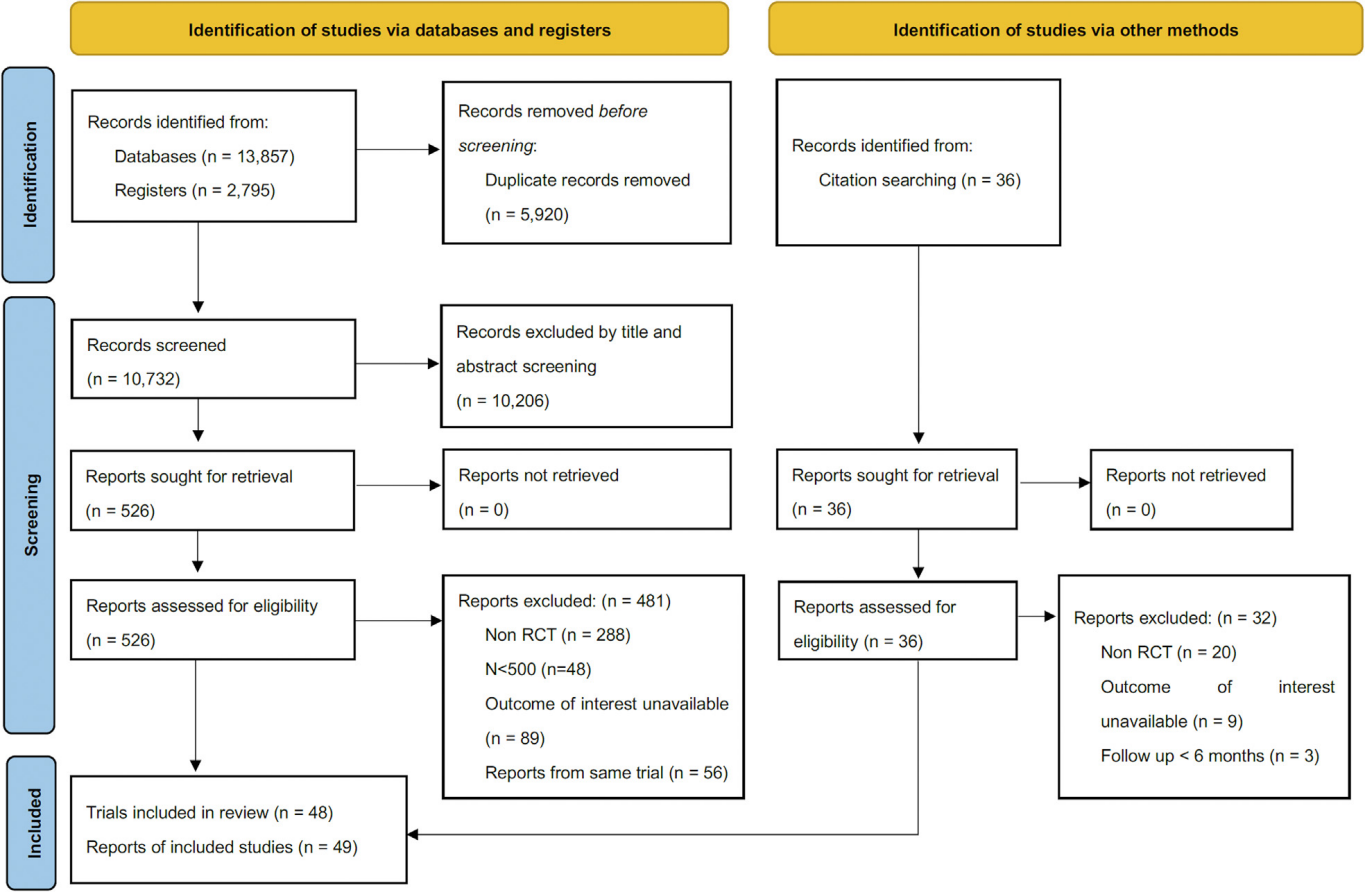
PRISMA Flowchart

We identified 48 trials that fulfilled inclusion criteria (Figure 1). Supplemental Table 4 provides characteristics of the 48 RCTs. Supplemental Table 5 shows the assessment of the risk of bias. I^2^ of odds ratio was 20.4%, 0.5 %, 90.7%, and 75.1% for all-cause mortality, cardiovascular mortality, trial defined major bleeding, and trial defined major/minor bleeding, respectively. Funnel plots is shown in Supplemental Figures 1-4. ORs of included studies of trial defined major/minor bleeding and major bleeding, and all-cause mortality is shown in Supplemental Figures 5 and 6. By the study period which we categorized by the first patient enrollment, 23 RCTs were initiated before 2010 and 25 RCTs after 2011. The follow-up duration was >12 months in 20 RCTs and ≤12 months in 28 RCTs (Table 1).

**Table 1:** Overall Analyses of the Correlation of Treatment Effects and Coefficient of Determination of Bleeding for All-Cause and Cardiovascular Mortality (OR)

Bleeding Criteria	Analysis	Comparison Pairs	RCTs	N	Regression Formula	Slope (95% CI)	R^2^ (95% CI)
Major or minor bleeding							
Study defined major or minor	All-cause mortality	43	35	136,560	−0.06 + 0.14∗log(OR_bleeding)	0.14 (0.00 to 0.28)	0.09 (0.00 to 0.26)
BARC 2,3,5	All-cause mortality	23	19	67,639	0.00 + 0.22∗log(OR_bleeding)	0.22 (0.01 to 0.43)	0.18 (0.00 to 0.48)
TIMI major or minor	All-cause mortality	19	17	74,701	−0.18 + 0.28∗log(OR_bleeding)	0.28 (0.14 to 0.43)	0.49 (0.14 to 0.84)
Study defined major or minor	CV mortality	40	33	124,830	−0.17 + 0.18∗log(OR_bleeding)	0.18 (0.00 to 0.35)	0.09 (0.00 to 0.27)
BARC 2,3,5	CV mortality	21	18	53,681	−0.13 + 0.16∗log(OR_bleeding)	0.16 (−0.16 to 0.48)	0.05 (0.00 to 0.26)
TIMI major or minor	CV mortality	18	16	70,701	−0.19 + 0.24∗log(OR_bleeding)	0.24 (−0.01 to 0.49)	0.21 (0.00 to 0.58)
Major bleeding							
Study defined major	All-cause mortality	53	44	188,773	−0.11 + 0.07∗log(OR_bleeding)	0.07 (−0.04 to 0.18)	0.03 (0.00 to 0.13)
BARC 3,5	All-cause mortality	22	19	70,813	−0.01 − 0.01∗log(OR_bleeding)	−0.01 (−0.26 to 0.23)	0.00 (0.00 to 0.02)
TIMI major	All-cause mortality	35	28	123,922	−0.19 + 0.08∗log(OR_bleeding)	0.08 (−0.03 to 0.19)	0.06 (0.00 to 0.23)
Study defined major	CV mortality	49	41	159,262	−0.17 + 0.03∗log(OR_bleeding)	0.03 (−0.09 to 0.15)	0.01 (0.00 to 0.05)
BARC 3,5	CV mortality	20	18	48,616	−0.09 − 0.11∗log(OR_bleeding)	−0.11 (−0.48 to 0.27)	0.02 (0.00 to 0.15)
TIMI major	CV mortality	32	26	110,750	−0.20 + 0.02∗log(OR_bleeding)	0.02 (−0.09 to 0.14)	0.01 (0.00 to 0.06)

BARC = Bleeding Academic Research Consortium; CV = cardiovascular; RCT = randomized controlled trial; TIMI = Thrombolysis In Myocardial Infarction.

### Coefficients of determination between treatment effects on bleeding outcomes and mortality

We plotted 1) the log-ORs for trial defined major/minor bleeding and those for all-cause mortality in 35 RCTs (43 comparisons) (Central Illustration A); and 2) the log-ORs for trial defined major/minor bleeding and those for cardiovascular mortality in 33 RCTs (40 comparisons) (Central Illustration B). The slope of the regression was 0.14 (95% CI: 0.00-0.28) in the regression of log-OR for all-cause mortality on log-OR for trial defined major/minor bleeding and 0.18 (95% CI: 0.00-0.35) in the regression of log-OR for cardiovascular mortality on log-OR for trial defined major/minor bleeding (Table 1). Both of trial-level coefficients of determination R^2^ did not reach the predefined threshold: 0.09 (95% CI: 0.00-0.26) between trial defined major/minor bleeding and all-cause mortality and 0.09 (95% CI: 0.00-0.27) between trial defined major/minor bleeding and cardiovascular mortality. In the analysis limiting RCTs which reported HR, we observed similar trial-level coefficients of determination R^2^ (Supplemental Table 6).

**Central Illustration:**
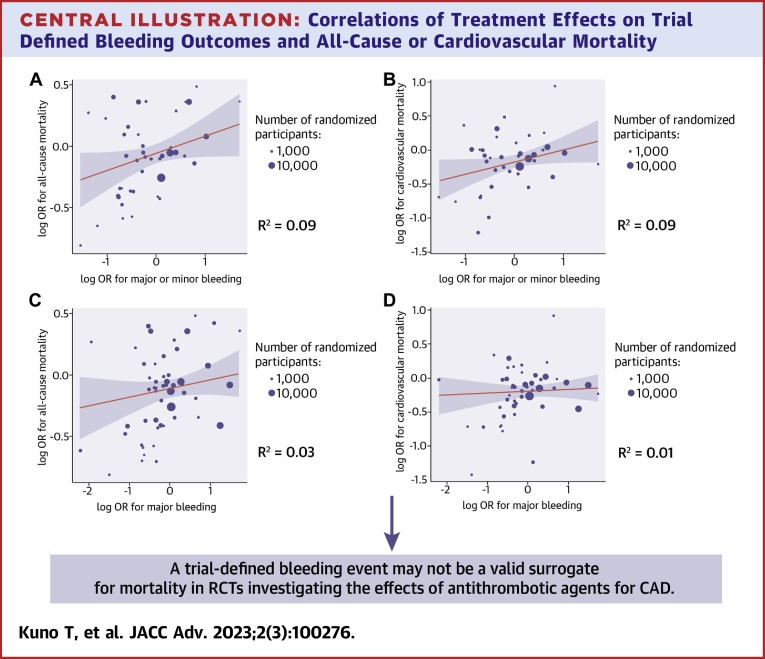
**Correlations of Treatment Effects on Trial Defined Bleeding Outcomes and All-Cause or Cardiovascular Mortality** **(A)** The association between the logarithm of the odds ratio (log OR) for trial defined major/minor bleeding (surrogate) and all-cause mortality. **(B)** The association between the logarithm of the odds ratio (log OR) for trial defined major/minor bleeding (surrogate) and cardiovascular mortality. **(C)** The association between the logarithm of the odds ratio (log OR) for trial defined major bleeding (surrogate) and all-cause mortality. **(D)** The association between the logarithm of the odds ratio (log OR) for trial defined major bleeding (surrogate) and cardiovascular mortality. **Circle sizes** are proportionate to the number of patients’ number (a total of intervention group and control group in each study).

Similarly, we plotted 1) the log-ORs for trial defined major bleeding and those for all-cause mortality in 44 RCTs (53 comparisons) (Central Illustration C); and 2) the log-ORs for trial defined major bleeding and those for cardiovascular mortality in 41 RCTs (49 comparisons) (Central Illustration D). The slope of the regression was 0.07 (95% CI: −0.04 to 0.18) in the regression of log-OR for all-cause mortality on log-OR for trial defined major bleeding and 0.03 (95% CI: −0.09 to 0.15) in the regression of log-OR for cardiovascular mortality on log-OR for trial defined major bleeding (Table 1). Both of trial-level coefficients of determination R^2^ did not reach the predefined threshold of 0.70: 0.03 (95% CI: 0.00-0.13) between trial defined major bleeding and all-cause mortality and 0.01 (95% CI: 0.00-0.05) between trial defined major bleeding and cardiovascular mortality. In the analysis limiting RCTs to those which reported HR, we observed similar trial-level coefficients of determination R^2^ (Supplemental Table 6).

In the subgroup analyses where we performed analyses for RCTs using BARC or TIMI bleeding criteria, the results remain similar (Table 1, Supplemental Table 6). Moreover, most of the subgroup analyses of study period, follow-up duration, trial locations mainly in East Asia or non-East Asia with major/minor or major bleeding in each definition for all-cause and cardiovascular mortality, did not reach significant trial-level coefficients of determination R^2^ as well as patients with acute coronary syndrome (Table 2, Supplemental Tables 7 to 14). One subgroup investigating the correlation of all-cause mortality and TIMI major or minor bleeding with the study follow-up period >12 months among all patients as well as patients with acute coronary syndrome showed R^2^ as 0.76 (95% CI: 0.61-1.00) and 0.89 (95% CI: 0.67-1.00), which did not meet the criteria of lower 95% CI as 0.72 as a significant correlation (Table 2, Supplemental Table 11). However, all-cause and cardiovascular mortality with TIMI major or minor bleeding among trials in East Asia showed R^2^ of 0.96 (95% CI: 0.81-1.00) and 0.98 (95% CI: 0.92-1.00), respectively, as strong correlations although there were only 4 trials included (Table 2 and Supplemental Table 8).

**Table 2:** All-Cause Mortality Major or Minor Bleeding Subanalysis Among Subgroups (OR)

	Comparison Pairs	RCTs	N	Regression Formula for All-Cause Mortality	Slope (95% CI)	R^2^ (95% CI)
Study defined major or minor bleeding						
Year of the first patient enrolment						
Before 2010	22	16	82,416	−0.15 + 0.26∗log(OR_bleeding)	0.26 (0.10 to 0.41)	0.37 (0.03 to 0.72)
After 2011	21	19	54,144	0.03 + 0.10∗log(OR_bleeding)	0.10 (−0.23 to 0.44)	0.02 (0.00 to 0.16)
Follow-up duration						
≤12 mo	27	23	69,446	−0.11 + 0.05∗log(OR_bleeding)	0.05 (−0.19 to 0.29)	0.01 (0.00 to 0.07)
>12 mo	16	12	67,114	−0.03 + 0.15∗log(OR_bleeding)	0.15 (−0.07 to 0.37)	0.14 (0.00 to 0.49)
Location of the trial						
East Asia	12	12	30,736	0.09 + 0.09∗log(OR_bleeding)	0.09 (−0.42 to 0.60)	0.01 (0.00 to 0.16)
Non-East Asia	31	23	105,824	−0.12 + 0.23∗log(OR_bleeding)	0.23 (0.09 to 0.36)	0.29 (0.01 to 0.58)
BARC 2, 3, or 5 bleeding						
Year of the first patient enrolment						
Before 2010	5	4	15,003	−0.17 + 0.45∗log(OR_bleeding)	0.45 (−1.01 to 1.90)	0.24 (0.00 to 1.00)
After 2011	18	15	52,636	0.00 + 0.20∗log(OR_bleeding)	0.20 (−0.16 to 0.57)	0.08 (0.00 to 0.35)
Follow-up duration						
≤12 mo	13	12	35,873	−0.05 + 0.12∗log(OR_bleeding)	0.12 (−0.20 to 0.45)	0.06 (0.00 to 0.34)
>12 mo	10	7	31,766	0.04 + 0.23∗log(OR_bleeding)	0.23 (−0.15 to 0.61)	0.19 (0.00 to 0.71)
Location of the trial						
East Asia	7	7	19,263	0.16 + 0.18∗log(OR_bleeding)	0.18 (−0.59 to 0.95)	0.07 (0.00 to 0.54)
Non-East Asia	16	12	48,376	−0.07 + 0.32∗log(OR_bleeding)	0.32 (0.11 to 0.53)	0.43 (0.03 to 0.84)
TIMI major or minor bleeding						
Year of the first patient enrolment						
Before 2010	13	11	61,202	−0.20 + 0.29∗log(OR_bleeding)	0.29 (0.13 to 0.44)	0.60 (0.22 to 0.98)
After 2011	6	6	13,499	−0.09 + 0.40∗log(OR_bleeding)	0.40 (−0.30 to 1.11)	0.39 (0.00 to 1.00)
Follow-up duration						
≤12 mo	11	10	37,257	−0.19 + 0.25∗log(OR_bleeding)	0.25 (−0.20 to 0.71)	0.15 (0.00 to 0.60)
>12 mo	8	7	37,444	−0.16 + 0.28∗log(OR_bleeding)	0.28 (0.16 to 0.39)	0.76 (0.61 to 1.00)
Location of the trial						
East Asia	4	4	7336	−0.09 + 0.59∗log(OR_bleeding)	0.59 (0.22 to 0.95)	0.96 (0.81 to 1.00)
Non-East Asia	15	13	67,365	−0.17 + 0.24∗log(OR_bleeding)	0.24 (0.07 to 0.41)	0.40 (0.00 to 0.83)

BARC = Bleeding Academic Research Consortium; CV = cardiovascular; RCT = randomized controlled trial; TIMI = Thrombolysis In Myocardial Infarction.

When we defined the different cutoff of R^2^ ≥0.70 as strong, between 0.50 and 0.69 as moderate, and <0.50 as a weak correlation, all of the main analyses were assessed as weak correlations.

## Discussion

In our meta-analysis, we demonstrated that the correlation of log-OR between trial defined major/minor or major bleeding and all-cause mortality in 48 RCTs (58 comparisons) were very low (0.09 and 0.03, respectively), implying that a bleeding outcome is not a valid surrogate for all-cause mortality in trials investigating antithrombotic agents for patients with CAD. Our results were consistent regardless of bleeding definitions, study period, follow-up period, and in patients with acute coronary syndrome.

Bleeding events are considered to be associated with death, especially with bleeding-related death that occurs within 30 days of the bleeding events.^17^^,^^24^^,^^35^ In comparison, bleeding events are typically not related to non-bleeding-related death.^24^ Our data investigating trial-level correlations between nonfatal bleeding events and mortality showed that a bleeding event cannot be a surrogate for death during the whole study period although bleeding events were associated with death shortly after the bleeding events.^24^ These findings should be informative for researchers who are considering using bleeding outcomes as a study end point to be a surrogate for death as well as to combine bleeding outcomes and death as NACE. Preferably, alternative analyses such as the win ratio analysis can be used to take into account for the priorities of the composite outcomes.^36^ In the TRILOGY-ACS and GLOBAL-LEADERS trials, trialists mentioned that various methods including the win ratio analysis should be considered to assess patients’ outcomes.^37^^,^^38^

NACE is frequently used as a study end point of antithrombotic agents for patients with CAD to assess the composite of major adverse cardiovascular and bleeding events.^18^, ^19^, ^20^ The TICO trial investigating short-term DAPT followed by ticagrelor vs ticagrelor-based 12-month DAPT for patients with acute coronary syndrome used NACE as the primary end point, which showed no significant difference in NACE between the 2 groups, but it was associated with higher bleeding events.^20^ Since NACE was even between the 2 groups as the primary end point, despite significantly different bleeding as the secondary outcome, the conclusion of the trial would be ambiguous. In general, physicians should select antithrombotic agents based on individualized ischemic and bleeding risks^39^; however, NACE as the primary end point may be inconclusive regarding which antithrombotic agents should be used for each patient. In addition, NACE has a risk of bias toward the null since thrombotic and bleeding outcomes often tend to go in opposite directions. Moreover, a composite outcome with a different magnitude of components may result in misleading impressions of the impact of treatments.^40^ Therefore, in isolation, NACE may not be an optimal trial primary endpoint since our data suggest bleeding events cannot be a surrogate for death.

Our study has several limitations. First, patient-level data were not available, and our study only included data available from the original papers and we could not assess HR in all enrolled studies. However, measures of correlation such as R^2^ or the slope of the regression the estimates of mortality on those of HF were similar among the analysis of log-ORs and log-HRs. Second, each study used a different definition of trial defined major/minor bleeding or major bleeding. Regardless, we also performed a subgroup analysis of BARC and TIMI bleeding criteria which remained similar to the analysis of trial defined bleeding outcomes. However, a subgroup of all-cause mortality and TIMI major/minor bleeding with follow-up period >12 months tend to have a correlation, which was the only exception in our results. Longer duration of the study with the use of TIMI major/minor bleeding could be a surrogate for mortality of patients with CAD. Nonetheless, the recent DAPT trials compare short DAPT vs standard DAPT as 12 months DAPT, which may not require long duration of follow-up >12 months because 12 months may be felt to be long enough to investigate the differences between short vs standard DAPT.^5^^,^^41^ Thus, less than one-half of the included trials did not have follow-up period >12 months, which can be a potential limitation of our study. Third, the relationship between the risk of bleeding and death may vary by race and region, especially in the East Asian population.^13^ We showed a potential correlation between TIMI major or minor bleeding and mortality among them; however, it should be interpreted cautiously because there were only 4 trials included in these analyses. Fourth, we investigated whether the correlation was linear or not. We could not exclude the possibility that the correlation is nonlinear. Finally, we showed bleeding events were not a surrogate for mortality; however, this does not mean bleeding events are not important for patients with CAD.

## Conclusions

Trial defined major or major/minor bleeding events may not be a valid surrogate for mortality. Assessments such as the win ratio may be preferable to NACE as the primary outcome in trials investigating antithrombotic agents for patients with CAD.

PERSPECTIVES **COMPETENCY IN MEDICAL KNOWLEDGE:** A bleeding event is not a valid surrogate for all-cause or cardiovascular death in trials investigating antithrombotic agents for patients with coronary artery disease.

**TRANSLATIONAL OUTLOOK:** Caution should be applied when choosing surrogate endpoints for all-cause and cardiovascular death. Assessments such as the win ratio may be preferable to NACE as the primary outcome in trials investigating antithrombotic agents for patients with coronary artery disease.

## Funding support and author disclosures

Dr Kohsaka has received a research grant for the Department of Cardiology, Keio University School of Medicine from Daiichi Sankyo Co., Ltd. but the funder did not have any role in the study design, data collection, data analysis, decision to publish, or manuscript preparation. Dr Slipczuk has received consulting honoraria from Amgen, Regeneron and Phillips and a research grant from 10.13039/100002429Amgen. Dr Bhatt is on the advisory board of AngioWave, Bayer, Boehringer Ingelheim, Cardax, CellProthera, Cereno Scientific, Elsevier Practice Update Cardiology, High Enroll, Janssen, Level Ex, McKinsey, Medscape Cardiology, Merck, MyoKardia, NirvaMed, Novo Nordisk, PhaseBio, PLx Pharma, Regado Biosciences, Stasys; Board of Directors: AngioWave (stock options), Boston VA Research Institute, Bristol Myers Squibb (stock), DRS.LINQ (stock options), High Enroll (stock), Society of Cardiovascular Patient Care, TobeSoft; Chair and Inaugural Chair of the American Heart Association Quality Oversight Committee; is a consultant for Broadview Ventures; Data Monitoring Committees: Acesion Pharma, Assistance Publique-Hôpitaux de Paris, Baim Institute for Clinical Research (formerly Harvard Clinical Research Institute, for the PORTICO trial, funded by 10.13039/100006279St. Jude Medical, now Abbott), Boston Scientific (Chair, PEITHO trial), Cleveland Clinic (including for the ExCEED trial, funded by 10.13039/100006520Edwards), Contego Medical (Chair, PERFORMANCE 2), Duke Clinical Research Institute, Mayo Clinic, Mount Sinai School of Medicine (for the ENVISAGE trial, funded by 10.13039/501100022274Daiichi Sankyo; for the ABILITY-DM trial, funded by Concept Medical), Novartis, Population Health Research Institute; Rutgers University (for the NIH-funded MINT Trial); has received honoraria from American College of Cardiology (Senior Associate Editor, Clinical Trials and News, ACC.org; is Chair of the ACC Accreditation Oversight Committee), Arnold and Porter law firm (work related to Sanofi/Bristol-Myers Squibb clopidogrel litigation), Baim Institute for Clinical Research (formerly Harvard Clinical Research Institute; is on the RE-DUAL PCI clinical trial steering committee funded by 10.13039/100001003Boehringer Ingelheim; AEGIS-II executive committee funded by 10.13039/100008322CSL Behring), Belvoir Publications (Editor in Chief, Harvard Heart Letter), Canadian Medical and Surgical Knowledge Translation Research Group (clinical trial steering committees), Cowen and Company, Duke Clinical Research Institute (clinical trial steering committees, including for the PRONOUNCE trial, funded by 10.13039/501100004914Ferring Pharmaceuticals), HMP Global (Editor in Chief, *Journal of Invasive Cardiology*), *Journal of the American College of Cardiology* (Guest Editor; Associate Editor), K2P (Co-Chair, interdisciplinary curriculum), Level Ex, Medtelligence/ReachMD (CME steering committees), MJH Life Sciences, Oakstone CME (Course Director, Comprehensive Review of Interventional Cardiology), Piper Sandler, Population Health Research Institute (for the COMPASS operations committee, publications committee, steering committee, and USA national co-leader, funded by 10.13039/100004326Bayer), Slack Publications (Chief Medical Editor, Cardiology Today’s Intervention), Society of Cardiovascular Patient Care (Secretary/Treasurer), WebMD (CME steering committees), Wiley (steering committee); is the Clinical Cardiology (Deputy Editor), NCDR-ACTION Registry Steering Committee (Chair), VA CART Research and Publications Committee (Chair); Patent: Sotagliflozin (named on a patent for sotagliflozin assigned to Brigham and Women's Hospital who assigned to Lexicon; neither I nor Brigham and Women's Hospital receive any income from this patent); has received research funding from 10.13039/100001316Abbott, Acesion Pharma, Afimmune, Aker Biomarine, 10.13039/100014384Amarin, 10.13039/100002429Amgen, 10.13039/100004325AstraZeneca, 10.13039/100004326Bayer, Beren, 10.13039/100001003Boehringer Ingelheim, 10.13039/100008497Boston Scientific, 10.13039/100002491Bristol-Myers Squibb, Cardax, CellProthera, Cereno Scientific, Chiesi, CinCor, 10.13039/100008322CSL Behring, 10.13039/501100003769Eisai, 10.13039/100009933Ethicon, Faraday Pharmaceuticals, 10.13039/501100004914Ferring Pharmaceuticals, 10.13039/100005632Forest Laboratories, Fractyl, Garmin, 10.13039/501100018679HLS Therapeutics, 10.13039/501100016198Idorsia, 10.13039/100010721Ironwood, Ischemix, 10.13039/100005565Janssen, Javelin, Lexicon, 10.13039/100004312Lilly, 10.13039/100004374Medtronic, 10.13039/100004334Merck, 10.13039/100019533Moderna, 10.13039/100016619MyoKardia, NirvaMed, 10.13039/100004336Novartis, 10.13039/501100004191Novo Nordisk, Owkin, 10.13039/100004319Pfizer, PhaseBio, PLx Pharma, Recardio, 10.13039/100009857Regeneron, Reid Hoffman Foundation, 10.13039/100004337Roche, 10.13039/100004339Sanofi, Stasys, Synaptic, The Medicines Company, Youngene, and 89Bio; has received royalties from Elsevier (Editor, Braunwald’s Heart Disease); Site Co-Investigator: Abbott, Biotronik, Boston Scientific, CSI, Endotronix, St. Jude Medical (now Abbott), Philips, SpectraWAVE, Svelte, Vascular Solutions; is a trustee with American College of Cardiology; and has received unfunded research from FlowCo and Takeda. All other authors have reported that they have no relationships relevant to the contents of this paper to disclose.

## References

[1] Kuno T., Numasawa Y., Sawano M. (2019). Real-world use of intravascular ultrasound in Japan: a report from contemporary multicenter PCI registry. Heart Vessels.

[2] Goryo Y., Kume T., Okamoto H. (2022). Influence of dual antiplatelet therapy duration on neointimal condition after second-generation drug-eluting stent implantation. Cardiovasc Interv Ther.

[3] Mehran R., Baber U., Sharma S.K. (2019). Ticagrelor with or without aspirin in high-risk patients after PCI. N Engl J Med.

[4] Watanabe H., Domei T., Morimoto T. (2019). Effect of 1-month dual antiplatelet therapy followed by clopidogrel vs 12-month dual antiplatelet therapy on cardiovascular and bleeding events in patients receiving PCI: the STOPDAPT-2 randomized clinical trial. JAMA.

[5] Watanabe H., Morimoto T., Natsuaki M. (2022). Comparison of clopidogrel monotherapy after 1 to 2 months of dual antiplatelet therapy with 12 months of dual antiplatelet therapy in patients with acute coronary syndrome: the STOPDAPT-2 ACS randomized clinical trial. JAMA Cardiol.

[6] Kuno T., Fujisaki T., Shoji S. (2022). Comparison of unguided de-escalation versus guided selection of dual antiplatelet therapy after acute coronary syndrome: a systematic review and network meta-analysis. Circ Cardiovasc Interv.

[7] Shoji S., Kuno T., Fujisaki T. (2021). De-escalation of dual antiplatelet therapy in patients with acute coronary syndromes. J Am Coll Cardiol.

[8] Yokoi H., Oda E., Kaneko K., Matsubayashi K. (2022). Duration and clinical outcome of dual antiplatelet therapy after percutaneous coronary intervention: a retrospective cohort study using a medical information database from Japanese hospitals. Cardiovasc Interv Ther.

[9] Watanabe H., Morimoto T., Ogita M. (2021). Influence of CYP2C19 genotypes for the effect of 1-month dual antiplatelet therapy followed by clopidogrel monotherapy relative to 12-month dual antiplatelet therapy on clinical outcomes after percutaneous coronary intervention: a genetic substudy from the STOPDAPT-2. Cardiovasc Interv Ther.

[10] Watanabe H., Domei T., Morimoto T. (2021). Details on the effect of very short dual antiplatelet therapy after drug-eluting stent implantation in patients with high bleeding risk: insight from the STOPDAPT-2 trial. Cardiovasc Interv Ther.

[11] Ozaki Y., Hara H., Onuma Y. (2022). CVIT expert consensus document on primary percutaneous coronary intervention (PCI) for acute myocardial infarction (AMI) update 2022. Cardiovasc Interv Ther.

[12] Liu P.Y., Su C.H., Kuo F.Y. (2022). Prasugrel switching from clopidogrel after percutaneous coronary intervention for acute coronary syndrome in Taiwanese patients: an analysis of safety and efficacy. Cardiovasc Interv Ther.

[13] Numasawa Y., Sawano M., Fukuoka R. (2020). Antithrombotic strategy for patients with acute coronary syndrome: a perspective from East Asia. J Clin Med.

[14] Kim C.J., Park M.W., Kim M.C. (2021). Unguided de-escalation from ticagrelor to clopidogrel in stabilised patients with acute myocardial infarction undergoing percutaneous coronary intervention (TALOS-AMI): an investigator-initiated, open-label, multicentre, non-inferiority, randomised trial. Lancet.

[15] Natsuaki M., Sonoda S., Yoshioka G. (2022). Antiplatelet therapy after percutaneous coronary intervention: current status and future perspectives. Cardiovasc Interv Ther.

[16] Ishida M., Takahashi F., Goto I. (2020). Clinical outcomes of patients treated using very short duration dual antiplatelet therapy after implantation of biodegradable-polymer drug-eluting stents: rationale and design of a prospective multicenter REIWA registry. Cardiovasc Interv Ther.

[17] Marquis-Gravel G., Dalgaard F., Jones A.D. (2020). Post-discharge bleeding and mortality following acute coronary syndromes with or without PCI. J Am Coll Cardiol.

[18] You S.C., Rho Y., Bikdeli B. (2020). Association of ticagrelor vs clopidogrel with net adverse clinical events in patients with acute coronary syndrome undergoing percutaneous coronary intervention. JAMA.

[19] Serruys P.W., Takahashi K., Chichareon P. (2019). Impact of long-term ticagrelor monotherapy following 1-month dual antiplatelet therapy in patients who underwent complex percutaneous coronary intervention: insights from the global leaders trial. Eur Heart J.

[20] Kim B.K., Hong S.J., Cho Y.H. (2020). Effect of ticagrelor monotherapy vs ticagrelor with aspirin on major bleeding and cardiovascular events in patients with acute coronary syndrome: the TICO randomized clinical trial. JAMA.

[21] Steg P.G., Bhatt D.L. (2018). Is there really a benefit to net clinical benefit in testing antithrombotics?. Circulation.

[22] Wittes J., Lakatos E., Probstfield J. (1989). Surrogate endpoints in clinical trials: cardiovascular diseases. Stat Med.

[23] Valgimigli M., Smits P.C., Frigoli E. (2022). Duration of antiplatelet therapy after complex percutaneous coronary intervention in patients at high bleeding risk: a MASTER DAPT trial sub-analysis. Eur Heart J.

[24] Palmerini T., Bacchi Reggiani L., Della Riva D. (2017). Bleeding-related deaths in relation to the duration of dual-antiplatelet therapy after coronary stenting. J Am Coll Cardiol.

[25] O'Fee K., Deych E., Ciani O., Brown D.L. (2021). Assessment of nonfatal myocardial infarction as a surrogate for all-cause and cardiovascular mortality in treatment or prevention of coronary artery disease: a meta-analysis of randomized clinical trials. JAMA Intern Med.

[26] Page M.J., McKenzie J.E., Bossuyt P.M. (2021). The PRISMA 2020 statement: an updated guideline for reporting systematic reviews. BMJ.

[27] Khan S.U., Singh M., Valavoor S. (2020). Dual antiplatelet therapy after percutaneous coronary intervention and drug-eluting stents: a systematic review and network meta-analysis. Circulation.

[28] Mehran R., Rao S.V., Bhatt D.L. (2011). Standardized bleeding definitions for cardiovascular clinical trials: a consensus report from the bleeding academic research consortium. Circulation.

[29] Bovill E.G., Terrin M.L., Stump D.C. (1991). Hemorrhagic events during therapy with recombinant tissue-type plasminogen activator, heparin, and aspirin for acute myocardial infarction. Results of the thrombolysis in myocardial infarction (TIMI), phase II trial. Ann Intern Med.

[30] Sterne J.A.C., Savović J., Page M.J. (2019). RoB 2: a revised tool for assessing risk of bias in randomised trials. BMJ.

[31] Xie W., Halabi S., Tierney J.F. (2019). A systematic review and recommendation for reporting of surrogate endpoint evaluation using meta-analyses. JNCI Cancer Spectr.

[32] Van der Elst W., Stijven F., Ong F. (2023). https://cran.r-project.org/web/packages/Surrogate/Surrogate.pdf.

[33] Buyse M., Molenberghs G., Burzykowski T., Renard D., Geys H. (2000). The validation of surrogate endpoints in meta-analyses of randomized experiments. Biostatistics.

[34] Buyse M., Molenberghs G., Paoletti X. (2016). Statistical evaluation of surrogate endpoints with examples from cancer clinical trials. Biom J.

[35] Genereux P., Giustino G., Witzenbichler B. (2015). Incidence, predictors, and impact of post-discharge bleeding after percutaneous coronary intervention. J Am Coll Cardiol.

[36] Redfors B., Gregson J., Crowley A. (2020). The win ratio approach for composite endpoints: practical guidance based on previous experience. Eur Heart J.

[37] Bakal J.A., Roe M.T., Ohman E.M. (2015). Applying novel methods to assess clinical outcomes: insights from the TRILOGY ACS trial. Eur Heart J.

[38] Hara H., van Klaveren D., Takahashi K. (2020). Comparative methodological assessment of the randomized GLOBAL LEADERS trial using total ischemic and bleeding events. Circ Cardiovasc Qual Outcomes.

[39] Valgimigli M., Bueno H., Byrne R.A. (2018). 2017 ESC focused update on dual antiplatelet therapy in coronary artery disease developed in collaboration with EACTS: the task force for dual antiplatelet therapy in coronary artery disease of the European Society of Cardiology (ESC) and of the European Association for Cardio-Thoracic Surgery (EACTS). Eur Heart J.

[40] Ferreira-Gonzalez I., Busse J.W., Heels-Ansdell D. (2007). Problems with use of composite end points in cardiovascular trials: systematic review of randomised controlled trials. BMJ.

[41] Hahn J.Y., Song Y.B., Oh J.H. (2019). Effect of P2Y12 Inhibitor monotherapy vs dual antiplatelet therapy on cardiovascular events in patients undergoing percutaneous coronary intervention: the SMART-CHOICE randomized clinical trial. JAMA.

