# Supplementary material for: Assessment of Nonfatal Bleeding Events as a Surrogate for Mortality in Coronary Artery Disease
Source: JACC Adv. 2023 Feb 20;2(3):100276. doi: 10.1016/j.jacadv.2023.100276 (PMC11198307; doi:10.1016/j.jacadv.2023.100276)
Supplement: Supplemental Figures 1-6 and Tables 1-14 [file mmc1.docx]

**Supplemental Figure 1.** Funnel plot for trial defined major or minor bleeding (a) and trial defined major bleeding (b)


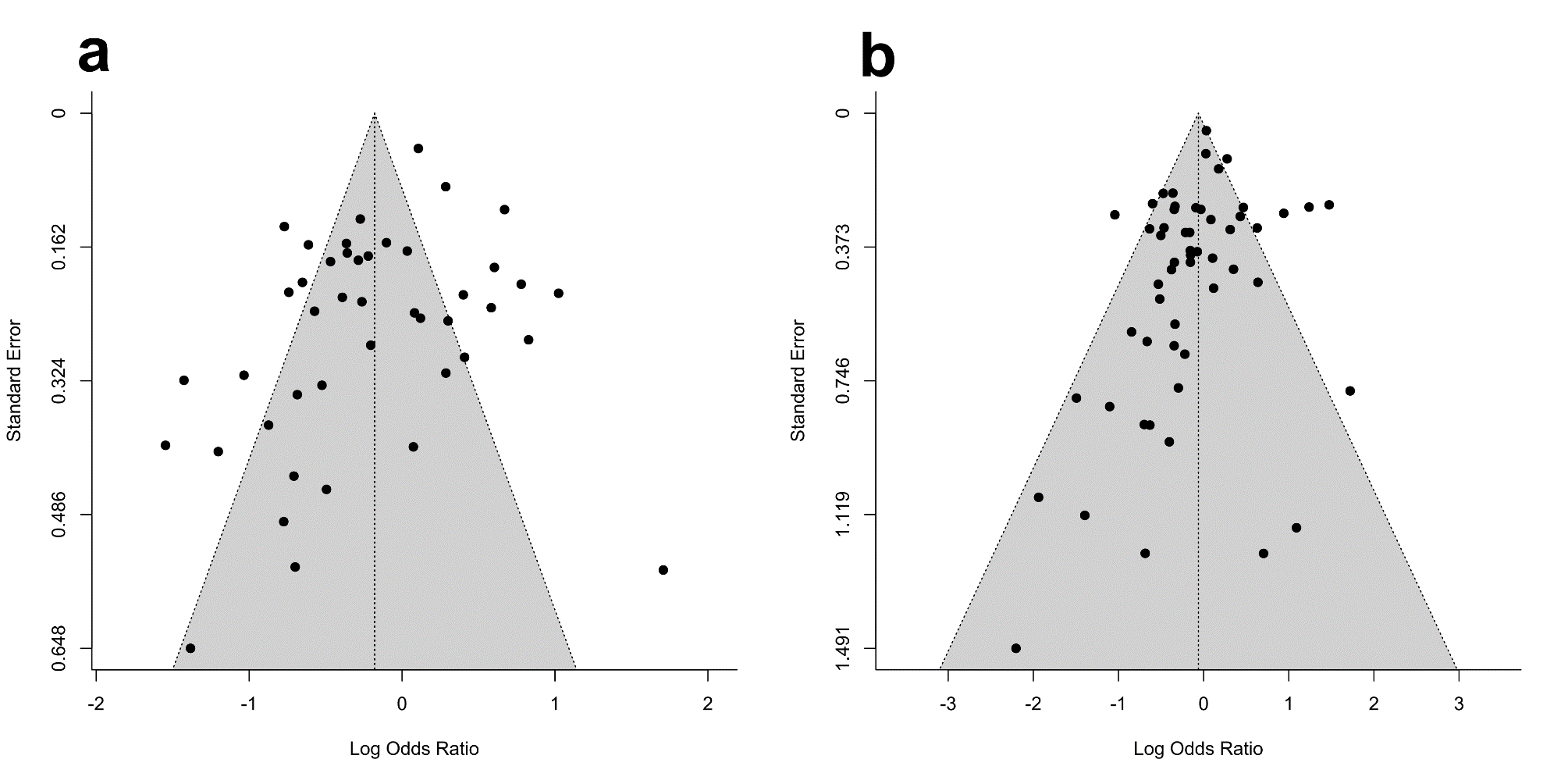


Significant publication bias was detected for trial defined major bleeding (p=.027) but not for trial defined major or minor bleeding (p=.051).

**Supplemental Figure 2.** Funnel plot for BARC 2, 3, or 5 bleeding (a) and BARC 3 or 5 bleeding (b)


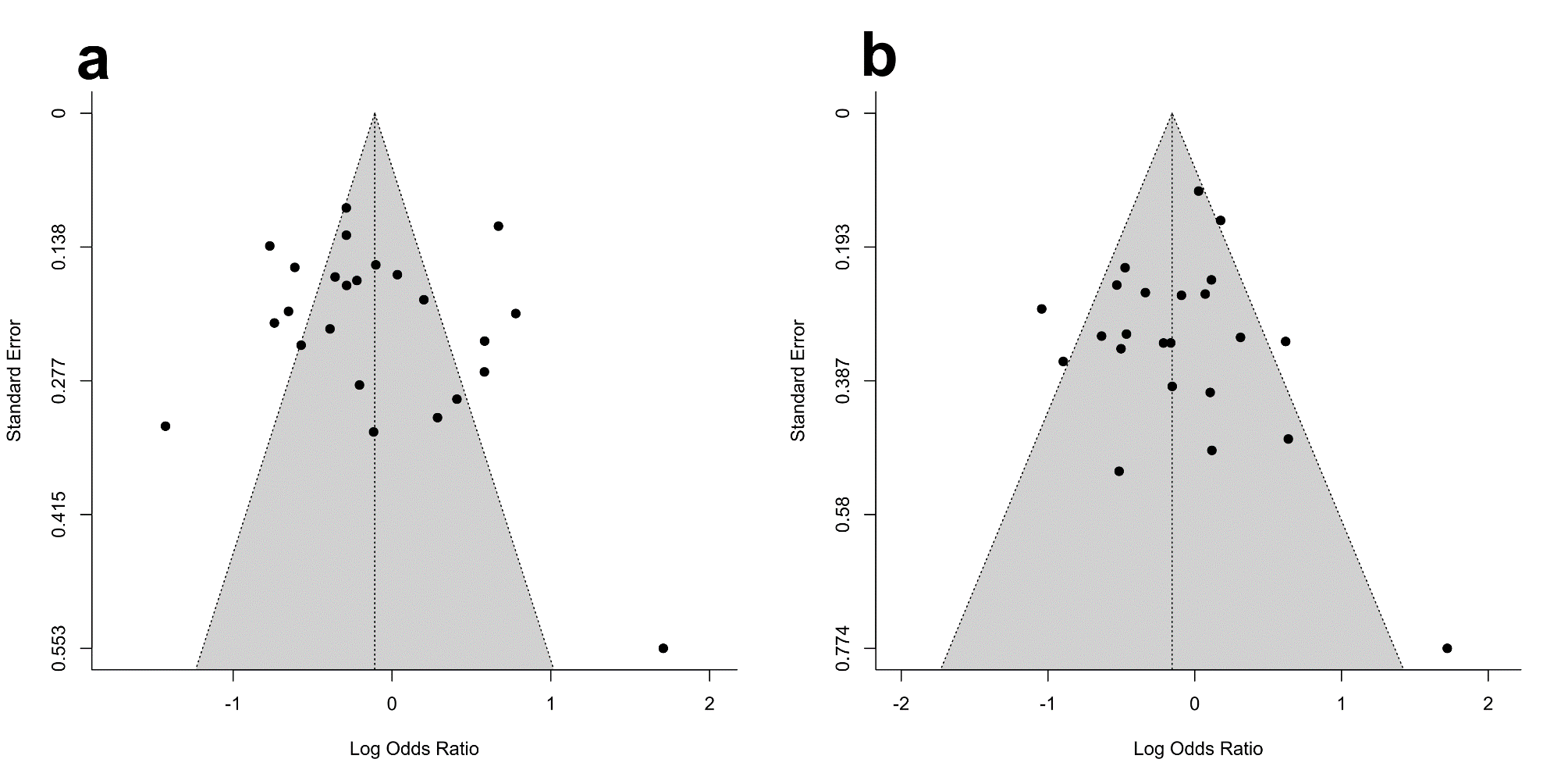


Significant publication bias was not detected for BARC 2, 3, or 5 bleeding (p=.107) nor BARC 3 or 5 bleeding (p=.316).

**Supplemental Figure 3.** Funnel plot for TIMI major or minor bleeding (a) and TIMI major bleeding (b)


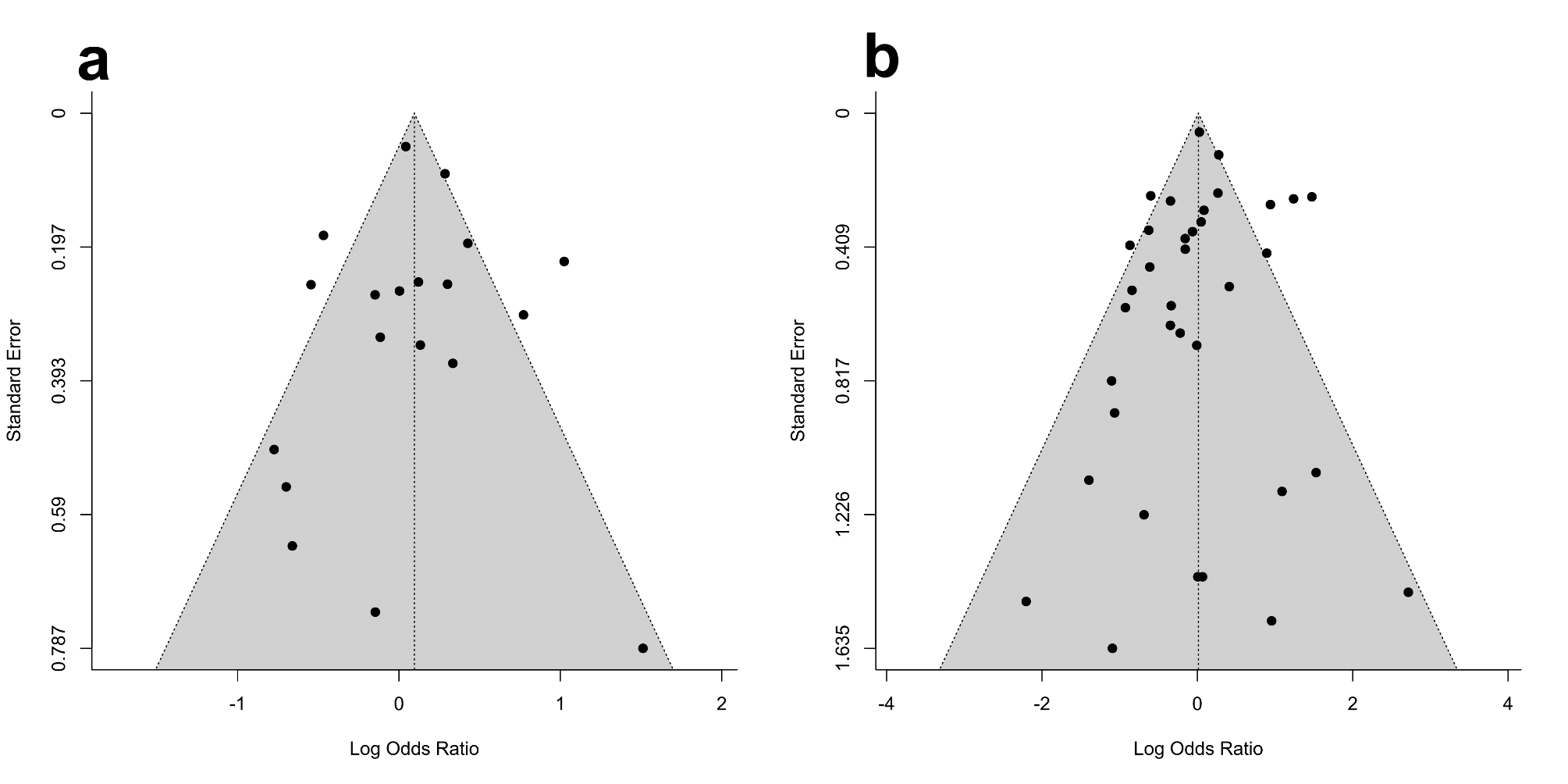


Significant publication bias was not detected for TIMI major or minor bleeding (p=.571) nor TIMI major bleeding (p=.294).

**Supplemental Figure 4.** Funnel plot for all-cause mortality (a) and cardiovascular mortality (b)


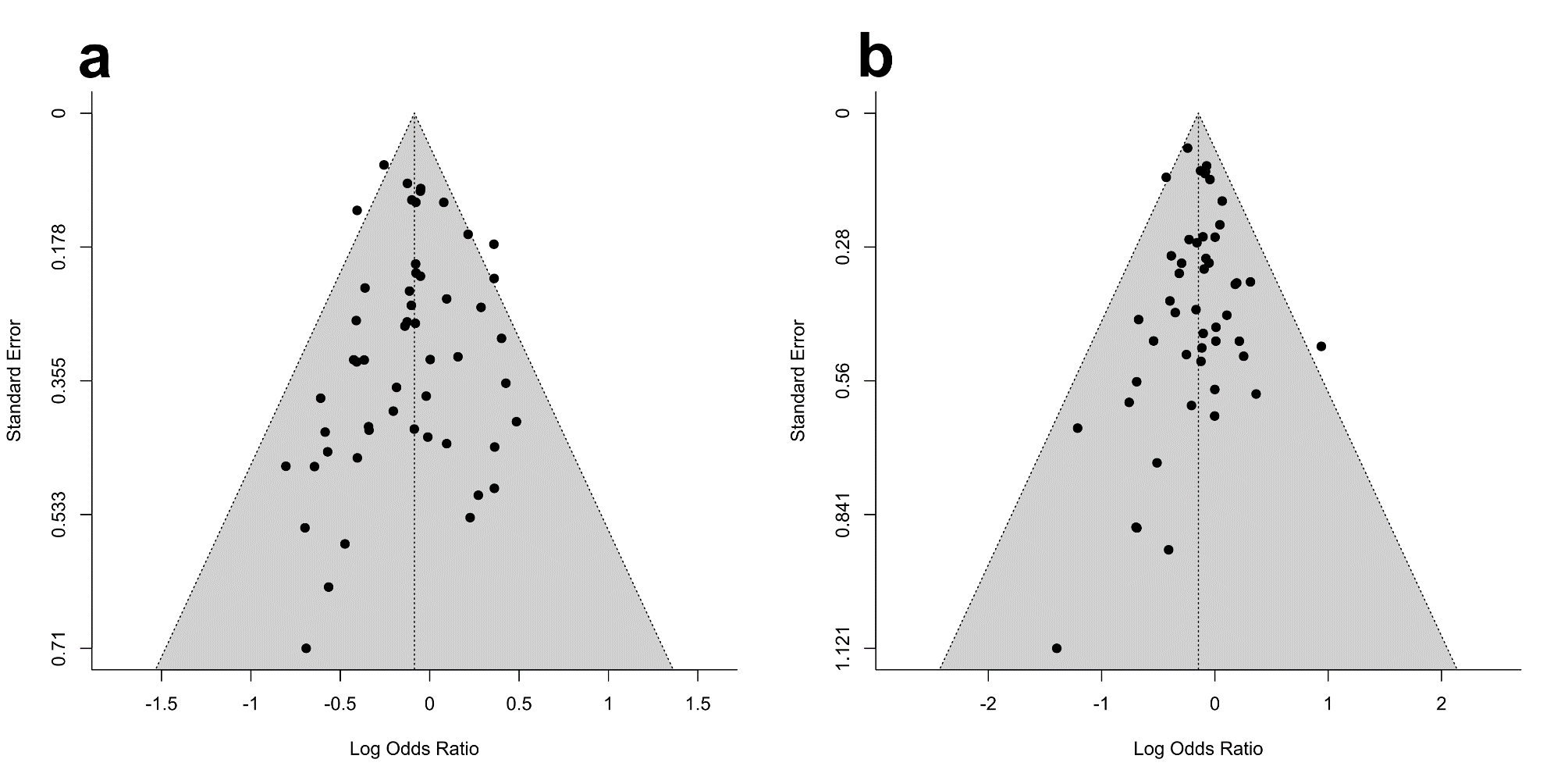


Significant publication bias was not detected for all-cause mortality (p=.488) nor cardiovascular mortality (p=.930).

**Supplemental Figure 5.** Treatment effect of trial defined major or minor bleeding and mortality


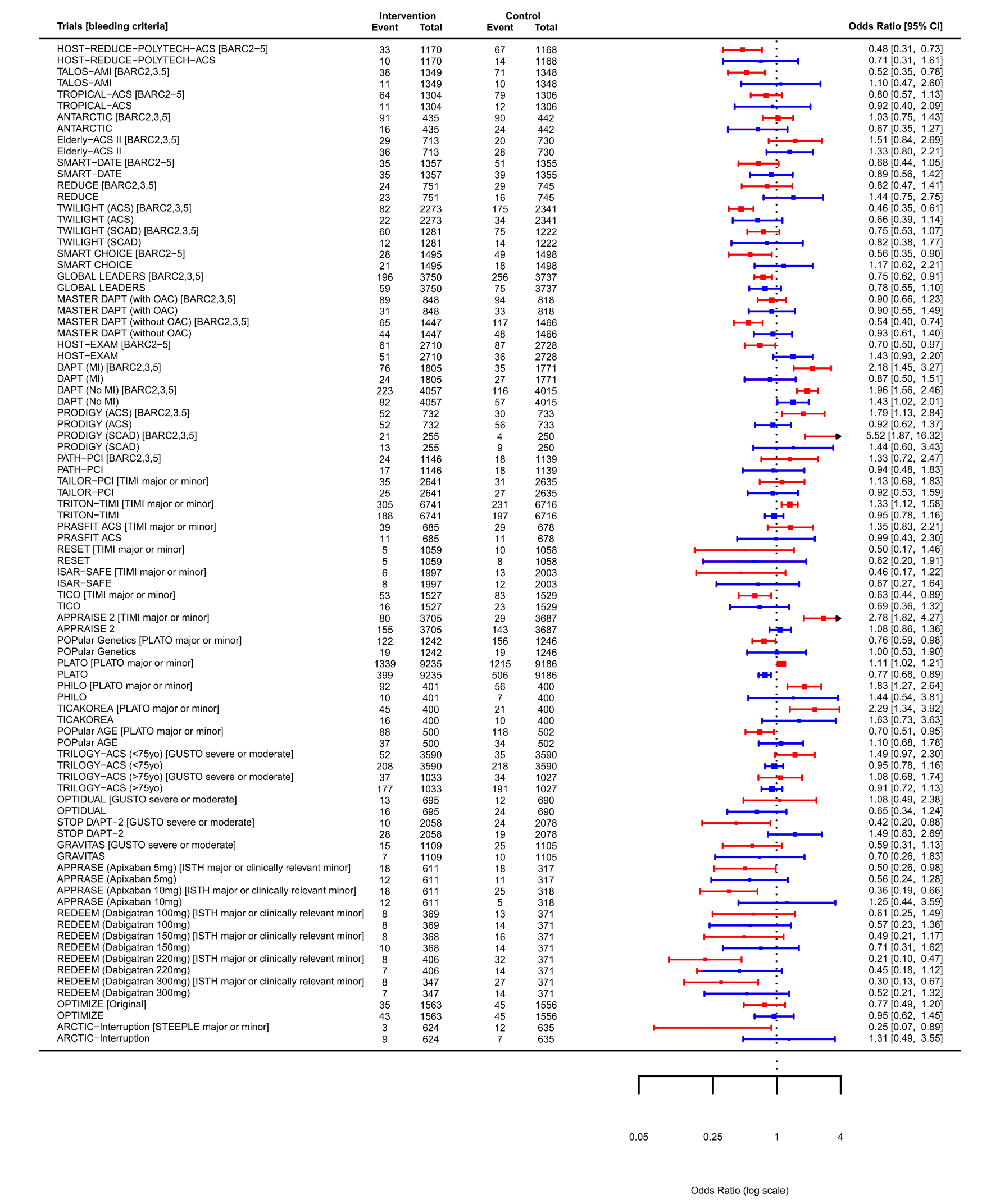


Red and blue denote odds ratio with 95% confidence interval for trial defined major/minor bleeding and all-cause mortality, respectively.

**Supplemental Figure 6**. Treatment effect of trial defined major or minor bleeding and mortality


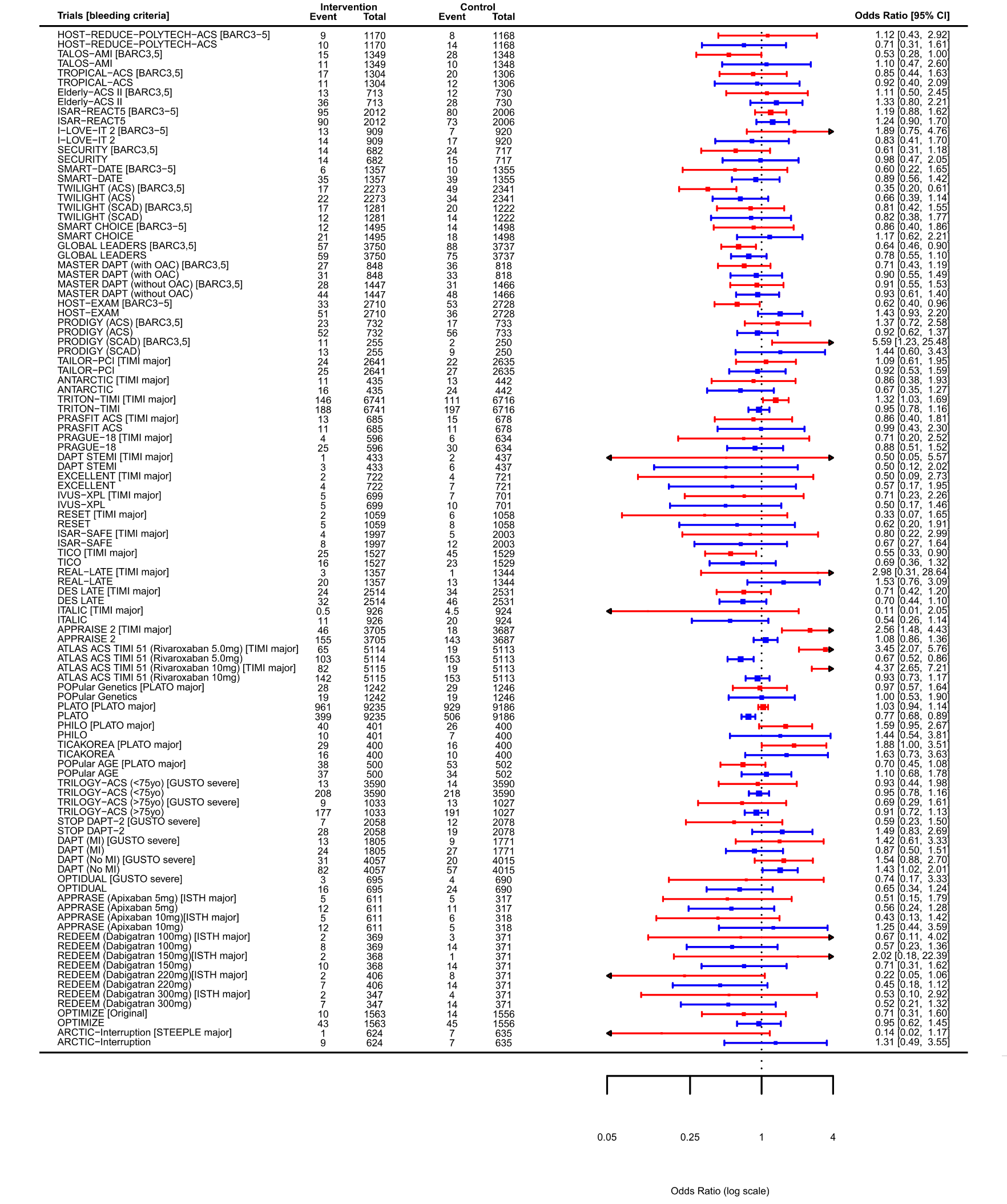


Red and blue denote odds ratio with 95% confidence interval for trial defined major bleeding and all-cause mortality, respectively.

**Supplemental Table 1.** The search strategy of MEDLINE via PubMed

| #1 | Percutaneous Coronary Intervention[mh] |
| --- | --- |
| #2 | "percutaneous coronary intervention"[tiab] |
| #3 | "PCI"[tiab] |
| #4 | "PTCA"[tiab] |
| #5 | #1 OR #2 OR #3 OR #4 |
| #6 | "dual antiplatelet therapy"[tiab] |
| #7 | "clopidogrel"[tiab] |
| #8 | "prasugrel"[tiab] |
| #9 | "ticagrelor"[tiab] |
| #10 | "p2y12" [tiab] |
| #11 | #6 OR #7 OR #8 OR #9 OR #10 |
| #12 | randomized controlled trial [pt] |
| #13 | controlled clinical trial [pt] |
| #14 | "randomized"[tiab] |
| #15 | "randomly"[tiab] |
| #16 | "placebo"[tiab] |
| #17 | #12 OR #13 OR #14 OR #15 OR #16 |
| #18 | #5 AND #11 AND #17 |

1977

**Supplemental Table 2.** The search strategy of Embase

| #1 | exp Ischemic heart disease/ or exp coronary artery/ or exp heart muscle ischemia/ or exp coronary artery disease/ |
| --- | --- |
| #2 | ("myocardial infarction" or "coronary artery" or "coronary syndrome" or "ischemic heart disease" or "coronary disease" or "Percutaneous coronary intervention" or "PCI" or "acute coronary syndromes" or "STEMI" or "unstable angina").ti,ab,kw. |
| #3 | antithrombotic agent/ or prasugrel/ or clopidogrel/ or ticagrelor/ |
| #4 | ("P2Y12 Inhibitor" or "Clopidogrel" or "ticagrelor" or "prasugrel" or "antiplatelet therapy" or "dual antiplatelet therapy" or "monotherapy").ti,ab,kw. |
| #5 | Randomized controlled trial/ or Controlled clinical study/ or Randomized/ |
| #6 | ("randomized controlled trial" or "controlled clinical trial").pt. or "randomized".ti,ab,kw. or "randomly".ti,ab,kw. |
| #7 | #1 or #2 |
| #8 | #3 or #4 |
| #9 | #5 or #6 |
| #10 | #7 and #8 and #9 |

11880

**Supplemental Table 3.** The search strategy of Cochran Central

| #1 | "Acute Coronary Syndrome" OR "Myocardial Infarction" OR "Coronary Thrombosis" OR "coronary disease" OR "acute coronary" OR "coronary syndrome" OR "ischemic heart disease" OR "Angina, Unstable" OR "acs" OR "ami" OR "acute angina" OR "unstable coronary" OR "stable coronary artery disease" OR "stable angina":ti,ab,kw |
| --- | --- |
| #2 | "Percutaneous Coronary Intervention" OR "PCI" OR "PTCA":ti,ab,kw |
| #3 | "dual antiplatelet therapy" OR "clopidogrel" OR "prasugrel" OR "ticagrelor" OR "p2y12" OR "monotherapy":ti,ab,kw |
| #5 | #1 and #2 and #3 |

2795

**Supplemental Table 4.** Trial characteristics

| **Trial name,** **publication date** | **Year of First Patient Enrollment** | **Region of the trial** | **Investigational arm** | **Control arm** | **Reported major or minor bleeding** | **Reported major bleeding** | **Investigational arm, N** | **Control arm, N** | **Median follow-up, months** |
| --- | --- | --- | --- | --- | --- | --- | --- | --- | --- |
| TOPIC, 2017 | 2014 | Europe | Unguided de-escalation of DAPT | 12-month DAPT with Ticagrelor or Prasugrel | BARC 2-5 | TIMI major | 323 | 323 | 12 |
| HOST-REDUCE-POLYTECH-ACS, 2020 | 2014 | East Asia | Unguided de-escalation of DAPT | 12-month DAPT with Prasugrel | BARC 2-5 | BARC 3-5 | 1,170 | 1,168 | 12 |
| TALOS-AMI, 2021 | 2014 | East Asia | Unguided de-escalation of DAPT | 12-month DAPT with Ticagrelor | BARC 2, 3, 5 | BARC 3, 5 | 1,349 | 1,348 | 12 |
| TROPICAL-ACS, 2017 | 2013 | Europe | Guided selection of P2y12i | 12-month DAPT with Prasugrel | BARC 2-5 | BARC 3-5 | 1,304 | 1,306 | 12 |
| ANTARCTIC, 2016 | 2012 | Europe | Guided selection of P2y12i | 12-month DAPT with low-dose Prasugrel | BARC 2, 3, 5; TIMI major or minor; GUSTO severe or moderate; STEEPLE major or minor; ISTH major or clinically relevant minor | TIMI major; GUSTO severe; STEEPLE major; ISTH major | 435 | 442 | 12 |
| Elderly-ACS II, 2018 | 2012 | Europe | 12-month DAPT with low-dose Prasugrel | 12-month DAPT with Clopidogrel | BARC 2, 3, 5 | BARC 3, 5 | 713 | 730 | 12 |
| ISAR-REACT 5, 2019 | 2013 | Europe | 12-month DAPT with Ticagrelor | 12-month DAPT with low-dose Prasugrel | - | BARC 3-5 | 2,012 | 2006 | 12 |
| I-LOVE-IT 2, 2016 | 2012 | East Asia | 6-month DAPT followed by Aspirin monotherapy | 12-month DAPT with Clopidogrel | - | BARC 3-5 | 909 | 920 | 18 |
| SMART-DATE, 2018 | 2012 | East Asia | 6-month DAPT followed by Aspirin monotherapy | 12-month DAPT with Clopidogrel | BARC 2-5 | BARC 3-5 | 1,357 | 1,355 | 18 |
| SECURITY, 2014 | 2009 | Europe | 6-month DAPT followed by Aspirin monotherapy | 12-month DAPT with Clopidogrel | - | BARC 3, 5 | 682 | 717 | 24 |
| REDUCE, 2019 | 2014 | Europe | 3-month DAPT followed by Aspirin monotherapy | 12-month DAPT with Ticagrelor | BARC 2, 3, 5 | - | 751 | 745 | 24 |
| TWILIGHT, 2020 | 2015 | North America | 3-month DAPT followed by Ticagrelor monotherapy | 15-month DAPT with Ticagrelor | BARC 2, 3, 5; GUSTO severe or moderate | BARC 3, 5; TIMI major; ISTH major | 3,554 | 3,563 | 15 |
| SMART CHOICE, 2019 | 2014 | East Asia | 3-month DAPT followed by Ticagrelor, Prasugrel, or Clopidogrel monotherapy | 12-month DAPT with Ticagrelor, Prasugrel, or Clopidogrel | BARC 2-5 | BARC 3-5 | 1,495 | 1,498 | 12 |
| GLOBAL LEADERS, 2018 | 2013 | Europe, North America, South America, Oceania, Southeast Asia, | 1-month DAPT followed by Ticagrelor monotherapy | 12-month DAPT with Ticagrelor | - | BARC 3, 5 | 7,980 | 7,988 | 24 |
| MASTER DAPT, 2021 | 2017 | Europe, South America, Oceania, Middle East, South Asia, Southeast Asia, East Asia | 5-month DAPT followed by Aspirin monotherapy | 12-month DAPT with Ticagrelor, Prasugrel, or Clopidogrel | BARC 2, 3, 5 | BARC 3, 5 | 2,295 | 2,284 | 12 |
| HOST-EXAM, 2021 | 2014 | East Aisa | 6-18-month DAPT followed by Clopidogrel monotherapy | 6-18-month DAPT followed by Aspirin monotherapy | BARC 2-5 | BARC 3-5 | 2,710 | 2,728 | 24 |
| DAPT, 2015 | 2009 | North America, Europe, Oceania | 30-month DAPT with Ticagrelor, Prasugrel, or Clopidogrel | 12-month DAPT with Ticagrelor, Prasugrel, or Clopidogrel | BARC 2, 3, 5 | GUSTO severe | 5,862 | 5,786 | 30 |
| PRODIGY, 2015 | 2006 | Europe | 24-month DAPT with Clopidogrel | 6-month DAPT followed by Aspirin monotherapy | BARC 2, 3, 5 | BARC 3, 5 | 987 | 983 | 24 |
| PATH-PCI, 2020 | 2016 | East Asia | Guided selection of P2y12i | 12-month DAPT with Clopidogrel | BARC 2, 3, 5 | - | 1,146 | 1,139 | 6 |
| PHARMCLO, 2018 | 2013 | Europe | Guided selection of P2y12i | 12-month DAPT with Clopidogrel | - | BARC 3-5 | 448 | 440 | 12 |
| TAILOR-PCI, 2020 | 2013 | North America, East Asia | Guided selection of P2y12i | 12-month DAPT with Clopidogrel | TIMI major or minor; BARC 2, 3, 5 | TIMI major; BARC 3, 5 | 2,641 | 2,635 | 12 |
| TRITON-TIMI, 2007 | 2004 | North America | 12-month DAPT with Prasugrel | 12-month DAPT with Clopidogrel | TIMI major or minor | TIMI major | 6,741 | 6,716 | 15 |
| PRASFIT ACS, 2014 | 2010 | East Asia | 12-month DAPT with low-dose Prasugrel | 12-month DAPT with Clopidogrel | TIMI major or minor | TIMI major | 685 | 678 | 12 |
| PRAGUE-18, 2018 | 2013 | Europe | 12-month DAPT with Ticagrelor | 12-month DAPT with Prasugrel | - | TIMI major | 596 | 634 | 12 |
| DAPT STEMI, 2018 | 2011 | Europe | 6-month DAPT followed by Aspirin monotherapy | 12-month DAPT with Ticagrelor, Prasugrel, or Clopidogrel | - | TIMI major | 433 | 437 | 24 |
| EXCELLENT, 2012 | 2008 | East Asia | 6-month DAPT followed by Aspirin monotherapy | 12-month DAPT with Clopidogrel | - | TIMI major | 722 | 721 | 12 |
| IVUS-XPL, 2016 | 2010 | East Asia | 6-month DAPT followed by Aspirin monotherapy | 12-month DAPT with Clopidogrel | - | TIMI major | 699 | 701 | 12 |
| RESET, 2012 | 2009 | East Asia | 3-month DAPT followed by Aspirin monotherapy | 12-month DAPT with Clopidogrel | TIMI major or minor | TIMI major | 1,059 | 1,058 | 12 |
| ISAR-SAFE, 2015 | 2006 | Europe, North America, East Asia | 6-month DAPT followed by Aspirin monotherapy | 12-month DAPT with Clopidogrel | TIMI major or minor | TIMI major | 794 | 807 | 15 |
| TICO, 2020 | 2015 | East Asia | 3-month DAPT followed by Ticagrelor monotherapy | 12-month DAPT with Ticagrelor | TIMI major or minor | TIMI major | 1,527 | 1,529 | 12 |
| REAL-LATE, 2010 | 2007 | East Asia | 24-month DAPT with Clopidogrel | 12-month DAPT with Clopidogrel | - | TIMI major | 1,357 | 1,344 | 24 |
| DES-LATE, 2014 | 2010 | East Asia | 12-month DAPT with Clopidogrel | 48-month DAPT with Clopidogrel | - | TIMI major | 2,514 | 2,531 | 36 |
| ITALIC, 2017 | 2008 | Europe | 6-month DAPT followed by Aspirin monotherapy | 24-month DAPT with Ticagrelor, Prasugrel, or Clopidogrel | - | TIMI major | 926 | 924 | 24 |
| APPRAISE 2, 2011 | 2009 | North America, South America, Oceania, Europe, East Asia, South Asia, Middle East, Southeast Asia, Africa | 24-month DAPT with Ticagrelor, Prasugrel, or Clopidogrel plus Apixaban | 12-month DAPT with Ticagrelor, Prasugrel, or Clopidogrel | TIMI major or minor; ISTH major or clinically relevant non-major; GUSTO severe or moderate | TIMI major; ISTH major; GUSTO severe | 3,705 | 3,687 | 15 |
| ATLAS-ACS-TIMI 51, 2012 | 2008 | North America, South America, Oceania, Europe, East Asia, South Asia, Middle East, Southeast Asia | 12-month DAPT with Ticagrelor, Prasugrel, or Clopidogrel plus Rivaroxaban | 12-month DAPT with Ticagrelor, Prasugrel, or Clopidogrel | - | TIMI major | 5,114 | 5,113 | 31 |
| POPular Genetics, 2019 | 2011 | Europe | Guided selection of P2y12i | 12-month DAPT with Ticagrelor | PLATO major or minor; BARC 2, 3, 5; TIMI major or minor | PLATO major; BARC 3-5; TIMI major | 1,242 | 1,246 | 12 |
| PLATO, 2009 | 2006 | North America, South America, Oceania, Europe, East Asia, South Asia, Southeast Asia, Middle East, Africa | 12-month DAPT with Ticagrelor | 12-month DAPT with Clopidogrel | PLATO major or minor; TIMI major or minor | PLATO major; TIMI major | 9,235 | 9,186 | 12 |
| PHILO, 2015 | 2011 | East Asia | 12-month DAPT with Ticagrelor | 12-month DAPT with Clopidogrel | PLATO major or minor | PLATO major | 401 | 400 | 12 |
| TICAKOREA, 2019 | 2014 | East Asia | 12-month DAPT with Ticagrelor | 12-month DAPT with Clopidogrel | PLATO major or minor; TIMI major or minor; BARC 2-5 | PLATO major; TIMI major; BARC 3-5 | 400 | 400 | 12 |
| POPular AGE, 2020 | 2013 | Europe | 12-month DAPT with Clopidogrel | 12-month DAPT with Ticagrelor | PLATO major or minor; TIMI major or minor | PLATO major; TIMI major; BARC 3, 5 | 500 | 502 | 12 |
| TRILOGY-ACS (<75yo), 2012 | 2008 | North America, South America, Oceania, East Asia, Africa, South Asia, Middle East, Southeast Asia | 12-month DAPT with Prasugrel | 12-month DAPT with Clopidogrel | GUSTO severe or moderate; TIMI major or minor | GUSTO severe; TIMI major | 3,590 | 3,590 | 30 |
| TRILOGY-ACS (>75yo), 2013 | 2008 | North America, South America, Oceania, East Asia, Africa, South Asia, Middle East, Southeast Asia | 12-month DAPT with low-dose Prasugrel | 12-month DAPT with Clopidogrel | GUSTO severe or moderate; TIMI major or minor | GUSTO severe; TIMI major | 1,033 | 1,027 | 30 |
| OPTIDUAL, 2016 | 2009 | Europe | 48-month DAPT with Clopidogrel | 12-month DAPT with Clopidogrel | GUSTO severe or moderate; BARC 2, 3, 5; TIMI major or minor | GUSTO severe; TIMI major; ISTH major | 695 | 690 | 48 |
| STOPDAPT-2 ACS, 2022 | 2015 | East Asia | 6-month DAPT followed by Clopidogrel or Prasugrel monotherapy | 12-month DAPT with Clopidogrel or Prasugrel | GUSTO severe or moderate | GUSTO severe; TIMI major; BARC 3, 5 | 2,058 | 2,078 | 12 |
| GRAVITAS, 2011 | 2008 | North America | 6-month DAPT with high-dose clopidogrel | 6-month DAPT with standard-dose clopidogrel | GUSTO severe or moderate | - | 1,109 | 1,105 | 6 |
| APPRAISE, 2009 | 2006 | North America, Europe, Middle East | 6-month DAPT with Clopidogrel plus Apixaban | 6-month DAPT with Clopidogrel | ISTH major or clinically relevant nonmajor | ISTH major | 635 | 611 | 6 |
| REDEEM, 2011 | 2008 | Europe | 6-month DAPT with Clopidogrel plus Dabigatran | 6-month DAPT with Clopidogrel | ISTH major or clinically relevant minor | ISTH major; TIMI major; GUSTO severe | 1,490 | 371 | 6 |
| OPTIMIZE, 2013 | 2010 | South America | 3-month DAPT followed by Aspirin monotherapy | 12-month DAPT with Clopidogrel | Bleeding that did not meet the criteria of GUSTO severe and REPLACE-2 | GUSTO severe and REPLACE-2 | 1,563 | 1,556 | 12 |
| ARCTIC-Interruption, 2014 | 2009 | Europe | 12-month DAPT with Clopidogrel or Prasugrel | 18-30-month DAPT with Clopidogrel or Prasugrel | STEEPLE major or minor | STEEPLE major | 624 | 635 | 24 |

**Supplemental Table 5.** Risk of bias of the included trials

| **Trial name, publication date** | **Random sequence generation (selection bias)** | **Allocation concealment (selection bias)** | **Blinding of participants and personnel (performance bias)** | **Blinding of outcome assessment (detection bias)** | **Incomplete outcome assessment (attrition bias)** | **Selective reporting (reporting bias)** | **Other bias** |
| --- | --- | --- | --- | --- | --- | --- | --- |
| TOPIC, 2017 | - | - | + | - | - | - | - |
| HOST-REDUCE-POLYTECH-ACS, 2020 | - | - | ? | - | - | - | - |
| TALOS-AMI, 2021 | - | - | + | - | ? | - | ? |
| TROPICAL-ACS, 2017 | ? | - | + | - | - | - | - |
| ANTARCTIC, 2016 | - | - | + | - | - | - | - |
| Elderly-ACS II, 2018 | - | - | ? | - | - | - | - |
| ISAR-REACT 5, 2019 | - | - | + | - | - | - | - |
| I-LOVE-IT 2, 2016 | + | ? | - | - | ? | - | ? |
| SMART-DATE, 2018 | - | + | - | - | - | - | - |
| SECURITY, 2014 | - | - | ? | ? | ? | - | - |
| REDUCE, 2019 | - | - | + | - | ? | - | - |
| TWILIGHT, 2020 | - | - | - | - | - | - | - |
| SMART CHOICE, 2019 | - | + | - | - | - | - | - |
| GLOBAL LEADERS, 2018 | - | + | - | - | - | - | - |
| MASTER DAPT, 2021 | - | - | + | - | - | - | - |
| HOST-EXAM, 2021 | - | - | + | - | - | - | - |
| DAPT, 2015 | - | - | - | - | + | - | - |
| PRODIGY, 2015 | - | - | ? | ? | + | - | - |
| PATH-PCI, 2020 | ? | ? | ? | ? | - | - | + |
| PHARMCLO, 2018 | - | - | + | - | - | - | - |
| TAILOR-PCI, 2020 | - | - | + | - | - | - | - |
| TRITON-TIMI, 2007 | - | - | - | - | - | - | - |
| PRASFIT ACS, 2014 | - | ? | - | - | - | - | - |
| PRAGUE-18, 2018 | - | - | + | - | - | - | - |
| DAPT STEMI, 2018 | - | - | + | - | - | - | - |
| EXCELLENT, 2012 | - | ? | - | - | + | - | - |
| IVUS-XPL, 2016 | - | ? | - | - | - | - | - |
| RESET, 2012 | - | ? | ? | - | - | - | - |
| ISAR-SAFE, 2015 | - | - | - | - | ? | - | - |
| TICO, 2020 | - | + | - | - | - | - | - |
| REAL-LATE, 2010 | - | - | - | - | - | - | - |
| DES-LATE, 2014 | - | + | - | - | + | - | - |
| ITALIC, 2017 | - | ? | ? | - | ? | - | - |
| APPRAISE 2, 2011 | - | - | - | - | - | - | - |
| ATLAS-ACS-TIMI 51, 2012 | - | - | - | - | - | - | - |
| POPular Genetics, 2019 | - | - | + | - | - | - | - |
| PLATO, 2009 | - | - | - | - | - | - | - |
| PHILO, 2015 | - | - | - | - | - | - | - |
| TICAKOREA, 2019 | - | - | + | - | - | - | - |
| POPular AGE, 2020 | - | - | + | - | - | - | - |
| TRILOGY-ACS (<75yo), 2012 | - | - | - | - | - | - | - |
| TRILOGY-ACS (>75yo), 2013 | - | - | - | - | - | - | - |
| OPTIDUAL, 2016 | - | - | ? | - | - | - | - |
| STOPDAPT-2 ACS, 2022 | - | - | - | - | - | - | - |
| GRAVITAS, 2011 | ? | - | ? | - | - | - | - |
| APPRAISE, 2009 | - | - | - | - | - | - | - |
| REDEEM, 2011 | - | - | - | - | - | - | - |
| OPTIMIZE, 2013 | - | - | - | - | - | - | - |
| ARCTIC-Interruption, 2014 | - | - | - | - | ? | - | - |

Each domain was judged as ‘low risk of bias’ (-), ‘high risk of bias’ (+), or ‘unclear risk of bias’ (?) in each study according to the Revised Cochrane risk-of-bias tool for randomized trials

**Supplemental table 6**. Overall analyses of the correlation of treatment effects and coefficient of determination of bleeding for all-cause and cardiovascular mortality (HR)

| Bleeding criteria | Analysis | Comparison pairs | RCTs | Patient number | Regression formula | Slope (95%CI) | R^2^ (95%CI) |
| --- | --- | --- | --- | --- | --- | --- | --- |
| Major or minor bleeding | | | | | | | |
| Study defined major or minor | All-cause mortality | 36 | 32 | 136,487 | -0.06+0.11*log(HR_bleeding) | 0.11 (-0.04 to 0.25) | 0.06 (0.00 to 0.21) |
| BARC 2,3,5 | All-cause mortality | 21 | 18 | 64,811 | 0.00+0.20*log(HR_bleeding) | 0.20 (-0.02 to 0.42) | 0.16 (0.00 to 0.47) |
| TIMI major or minor | All-cause mortality | 16 | 15 | 71,558 | -0.17+0.28*log(HR_bleeding) | 0.28 (0.15 to 0.41) | 0.60 (0.27 to 0.94) |
| Study defined major or minor | CV mortality | 31 | 28 | 114,214 | -0.14+0.13*log(HR_bleeding) | 0.13 (-0.08 to 0.34) | 0.05 (0.00 to 0.21) |
| BARC 2,3,5 | CV mortality | 19 | 17 | 50,853 | -0.12+0.15*log(HR_bleeding) | 0.15 (-0.20 to 0.50) | 0.05 (0.00 to 0.25) |
| TIMI major or minor | CV mortality | 14 | 13 | 64,502 | -0.14+0.13*log(HR_bleeding) | 0.13 (-0.12 to 0.38) | 0.10 (0.00 to 0.43) |
| Major bleeding | | | | | | | |
| Study defined Major | All-cause mortality | 42 | 37 | 166,992 | -0.09+0.04*log(HR_bleeding) | 0.04 (-0.09 to 0.16) | 0.01 (0.00 to 0.07) |
| BARC 3,5 | All-cause mortality | 20 | 17 | 59,060 | 0.01+0.07*log(HR_bleeding) | 0.07 (-0.21 to 0.36) | 0.02 (0.00 to 0.13) |
| TIMI Major | All-cause mortality | 24 | 22 | 112,004 | -0.17+0.08*log(HR_bleeding) | 0.08 (-0.07 to 0.22) | 0.05 (0.00 to 0.23) |
| Study defined Major | CV mortality | 34 | 30 | 136,601 | -0.16+0.00*log(HR_bleeding) | 0.00 (-0.15 to 0.15) | 0.00 (0.00 to 0.00) |
| BARC 3,5 | CV mortality | 15 | 12 | 39,039 | -0.12-0.13*log(HR_bleeding) | -0.13 (-0.64 to 0.37) | 0.02 (0.00 to 0.20) |
| TIMI Major | CV mortality | 19 | 18 | 95,776 | -0.18+0.04*log(HR_bleeding) | 0.04 (-0.12 to 0.19) | 0.01 (0.00 to 0.12) |

*BARC*, bleeding academic research consortium; *CI*, confidence interval; *CV*, cardiovascular; *HR*, hazard ratio; *RCT*, randomized controlled trial; *TIMI*, thrombolysis in myocardial infarction;

**Supplemental table 7**. All-cause mortality and major bleeding sub-analysis (OR)

| Analysis |  | Comparison pairs | RCTs | Patient number | Regression formula for all-cause mortality | Slope (95%CI) | R^2^ (95%CI) |
| --- | --- | --- | --- | --- | --- | --- | --- |
| Study defined Major bleeding | | | | | | | |
| Year of the first patient enrollment | Before 2010 | 29 | 22 | 114,495 | -0.17+0.11*log(OR_bleeding) | 0.11 (-0.02 to 0.23) | 0.10 (0.00 to 0.32) |
|  | After 2011 | 24 | 22 | 74,278 | -0.02+0.11*log(OR_bleeding) | 0.11 (-0.18 to 0.40) | 0.03 (0.00 to 0.16) |
| Follow-up duration | ≤12 months | 29 | 24 | 73,038 | -0.08+0.20*log(OR_bleeding) | 0.20 (-0.09 to 0.48) | 0.07 (0.00 to 0.25) |
|  | >12 months | 24 | 20 | 115,735 | -0.10+0.05*log(OR_bleeding) | 0.05 (-0.09 to 0.20) | 0.03 (0.00 to 0.16) |
| Location of the trial | East Asia | 16 | 16 | 40,869 | 0.03+0.22*log(OR_bleeding) | 0.22 (-0.15 to 0.60) | 0.10 (0.00 to 0.42) |
|  | Non-East Asia | 37 | 28 | 147,904 | -0.14+0.07*log(OR_bleeding) | 0.07 (-0.04 to 0.18) | 0.05 (0.00 to 0.19) |
| BARC 3 or 5 bleeding | | | | | | | |
| Year of the first patient enrollment | Before 2010 | 3 | 2 | 3,369 | -0.02+0.15*log(OR_bleeding) | 0.15 (-1.51 to 1.82) | 0.58 (0.00 to 1.00) |
|  | After 2011 | 19 | 17 | 67,444 | -0.02-0.04*log(OR_bleeding) | -0.04 (-0.33 to 0.25) | 0.01 (0.00 to 0.08) |
| Follow-up duration | ≤12 months | 13 | 12 | 34,380 | 0.04-0.21*log(OR_bleeding) | -0.21 (-0.55 to 0.13) | 0.14 (0.00 to 0.55) |
|  | >12 months | 9 | 7 | 36,433 | -0.07+0.09*log(OR_bleeding) | 0.09 (-0.31 to 0.49) | 0.04 (0.00 to 0.34) |
| Location of the trial | East Asia | 8 | 8 | 22,943 | 0.04-0.30*log(OR_bleeding) | -0.30 (-0.79 to 0.19) | 0.27 (0.00 to 0.94) |
|  | Non-East Asia | 14 | 11 | 47,870 | -0.06+0.29*log(OR_bleeding) | 0.29 (0.10 to 0.47) | 0.49 (0.07 to 0.91) |
| TIMI Major bleeding | | | | | | | |
| Year of the first patient enrollment | Before 2010 | 24 | 18 | 97,070 | -0.24+0.11*log(OR_bleeding) | 0.11 (-0.01 to 0.23) | 0.14 (0.00 to 0.42) |
|  | After 2011 | 11 | 10 | 26,852 | -0.03+0.21*log(OR_bleeding) | 0.21 (-0.34 to 0.76) | 0.08 (0.00 to 0.43) |
| Follow-up duration | ≤12 months | 19 | 15 | 48,440 | -0.21-0.09*log(OR_bleeding) | -0.09 (-0.39 to 0.21) | 0.02 (0.00 to 0.17) |
|  | >12 months | 16 | 13 | 75,482 | -0.22+0.13*log(OR_bleeding) | 0.13 (-0.01 to 0.26) | 0.22 (0.00 to 0.61) |
| Location of the trial | East Asia | 9 | 9 | 22,061 | 0.00+0.37*log(OR_bleeding) | 0.37 (-0.11 to 0.84) | 0.32 (0.00 to 0.94) |
|  | Non-East Asia | 26 | 19 | 101,861 | -0.21+0.06*log(OR_bleeding) | 0.06 (-0.04 to 0.17) | 0.06 (0.00 to 0.25) |

*BARC*, bleeding academic research consortium; *CI*, confidence interval; *CV*, cardiovascular; *OR*, odds ratio; *RCT*, randomized controlled trial; *TIMI*, thrombolysis in myocardial infarction;

**Supplemental table 8**. Cardiovascular mortality and major or minor bleeding sub-analysis (OR)

| Analysis |  | Comparison pairs | RCTs | Patient number | Regression formula for CV mortality | Slope (95%CI) | R^2^ (95%CI) |
| --- | --- | --- | --- | --- | --- | --- | --- |
| Study defined major or minor bleeding | | | | | | | |
| Year of the first patient enrollment | Before 2010 | 20 | 14 | 77,157 | -0.24+0.24*log(OR_bleeding) | 0.24 (0.04 to 0.43) | 0.27 (0.00 to 0.63) |
|  | After 2011 | 20 | 19 | 47,673 | -0.03+0.32*log(OR_bleeding) | 0.32 (-0.14 to 0.79) | 0.10 (0.00 to 0.38) |
| Follow-up duration | ≤12 months | 28 | 23 | 70.092 | -0.18+0.29*log(OR_bleeding) | 0.29 (0.00 to 0.59) | 0.14 (0.00 to 0.39) |
|  | >12 months | 12 | 10 | 54,738 | 0.00-0.14*log(OR_bleeding) | -0.14 (-0.43 to 0.15) | 0.10 (0.00 to 0.48) |
| Location of the trial | East Asia | 12 | 12 | 30,736 | -0.02+0.38*log(OR_bleeding) | 0.38 (-0.35 to 1.12) | 0.12 (0.00 to 0.51) |
|  | Non-East Asia | 28 | 21 | 94,094 | -0.19+0.17*log(OR_bleeding) | 0.17 (0.00 to 0.35) | 0.13 (0.00 to 0.38) |
| BARC 2,3, or 5 bleeding | | | | | | | |
| Year of the first patient enrollment | Before 2010 | 5 | 3 | 15,003 | -0.15+0.06*log(OR_bleeding) | 0.06 (-1.11 to 1.22) | 0.01 (0.00 to 0.26) |
|  | After 2011 | 16 | 16 | 38,678 | -0.03+0.39*log(OR_bleeding) | 0.39 (-0.20 to 0.99) | 0.13 (0.00 to 0.46) |
| Follow-up duration | ≤12 months | 13 | 12 | 29,032 | -0.13+0.44*log(OR_bleeding) | 0.44 (-0.11 to 1.00) | 0.22 (0.00 to 0.67) |
|  | >12 months | 8 | 7 | 24,649 | 0.05-0.20*log(OR_bleeding) | -0.20 (-0.67 to 0.27) | 0.15 (0.00 to 0.73) |
| Location of the trial | East Asia | 7 | 7 | 19,263 | 0.02+0.46*log(OR_bleeding) | 0.46 (-1.17 to 2.08) | 0.09 (0.00 to 0.64) |
|  | Non-East Asia | 14 | 11 | 34,418 | -0.14+0.13*log(OR_bleeding) | 0.13 (-0.18 to 0.43) | 0.06 (0.00 to 0.34) |
| TIMI major or minor bleeding | | | | | | | |
| Year of the first patient enrollment | Before 2010 | 12 | 9 | 57,202 | -0.21+0.21*log(OR_bleeding) | 0.21 (-0.05 to 0.46) | 0.24 (0.00 to 0.73) |
|  | After 2011 | 6 | 6 | 13,499 | -0.5+0.78*log(OR_bleeding) | 0.78 (-0.15 to 1.71) | 0.58 (0.00 to 1.00) |
| Follow-up duration | ≤12 months | 11 | 10 | 37,257 | -0.16+0.56*log(OR_bleeding) | 0.56(-0.07 to 1.18) | 0.31 (0.00 to 0.85) |
|  | >12 months | 7 | 6 | 33,444 | -0.14+0.09*log(OR_bleeding) | 0.09 (-0.09 to 0.27) | 0.24 (0.00 to 0.97) |
| Location of the trial | East Asia | 4 | 4 | 7,336 | -0.01+1.06*log(OR_bleeding) | 1.06 (0.64 to 1.49) | 0.98 (0.92 to 1.00) |
|  | Non-East Asia | 14 | 12 | 63,365 | -0.14+0.05*log(OR_bleeding) | 0.05 (-0.17 to 0.28) | 0.02 (0.00 to 0.20) |

*BARC*, bleeding academic research consortium; *CI*, confidence interval; *CV*, cardiovascular; *OR*, odds ratio; *RCT*, randomized controlled trial; *TIMI*, thrombolysis in myocardial infarction;

**Supplemental table 9**. Cardiovascular mortality and major bleeding sub-analysis (OR)

| Analysis |  | Comparison pairs | RCTs | Patient number | Regression of formula for CV mortality | Slope (95%CI) | R^2^ (95%CI) |
| --- | --- | --- | --- | --- | --- | --- | --- |
| Study defined Major bleeding | | | | | | | |
| Year of the first patient enrollment | Before 2010 | 26 | 19 | 106,535 | -0.20+0.03*log(OR_bleeding) | 0.03 (-0.08 to 0.14) | 0.01 (0.00 to 0.10) |
|  | After 2011 | 23 | 22 | 52,727 | -0.09+0.20*log(OR_bleeding) | 0.20 (-0.25 to 0.65) | 0.04 (0.00 to 0.20) |
| Follow-up duration | ≤12 months | 31 | 26 | 74,572 | -0.15+0.31*log(OR_bleeding) | 0.31 (0.01 to 0.62) | 0.13 (0.00 to 0.37) |
|  | >12 months | 18 | 15 | 84,690 | -0.11-0.06*log(OR_bleeding) | -0.06 (-0.20 to 0.08) | 0.05 (0.00 to 0.27) |
| Location of the trial | East Asia | 15 | 15 | 38,168 | -0.15+0.15*log(OR_bleeding) | 0.15 (-0.49 to 0.79) | 0.02 (0.00 to 0.17) |
|  | Non-East Asia | 34 | 26 | 121,094 | -0.16+0.01*log(OR_bleeding) | 0.01 (-0.10 to 0.11) | 0.00 (0.00 to 0.02) |
| BARC 3 or 5 bleeding | | | | | | | |
| Year of the first patient enrollment | Before 2010 | 3 | 2 | 3,369 | 0.08-0.18*log(OR_bleeding) | -0.18 (-0.48 to 0.13) | 0.98 (0.00 to 1.00) |
|  | After 2011 | 17 | 16 | 45,247 | -0.11-0.12*log(OR_bleeding) | -0.12 (-0.59 to 0.36) | 0.02 (0.00 to 0.16) |
| Follow-up duration | ≤12 months | 14 | 13 | 35,268 | -0.14-0.06*log(OR_bleeding) | -0.06 (-0.64 to 0.53) | 0.00 (0.00 to 0.07) |
|  | >12 months | 6 | 5 | 13,348 | 0.04-0.16*log(OR_bleeding) | -0.16 (-0.72 to 0.39) | 0.14 (0.00 to 0.88) |
| Location of the trial | East Asia | 8 | 8 | 22,943 | -0.15-0.21*log(OR_bleeding) | -0.21 (-1.20 to 0.78) | 0.04 (0.00 to 0.39) |
|  | Non-East Asia | 12 | 10 | 25,673 | -0.07-0.01*log(OR_bleeding) | -0.01 (-0.29 to 0.27) | 0.00 (0.00 to 0.04) |
| TIMI Major bleeding | | | | | | | |
| Year of the first patient enrollment | Before 2010 | 22 | 17 | 90,369 | -0.21+0.00*log(OR_bleeding) | 0.00 (-0.11 to 0.12) | 0.00 (0.00 to 0.01) |
|  | After 2011 | 10 | 10 | 20,381 | 0.05+0.58*log(OR_bleeding) | 0.58 (0.06 to 1.11) | 0.45 (0.00 to 1.00) |
| Follow-up duration | ≤12 months | 20 | 16 | 49,086 | -0.20+0.10*log(OR_bleeding) | 0.10 (-0.21 to 0.40) | 0.02 (0.00 to 0.17) |
|  | >12 months | 12 | 11 | 61,664 | -0.18-0.01*log(OR_bleeding) | -0.01 (-0.15 to 0.13) | 0.00 (0.00 to 0.06) |
| Location of the trial | East Asia | 8 | 8 | 19,360 | 0.07+0.67*log(OR_bleeding) | 0.67 (0.03 to 1.32) | 0.52 (0.00 to 1.00) |
|  | Non-East Asia | 24 | 18 | 91,390 | -0.16-0.03*log(OR_bleeding) | -0.03 (-0.15 to 0.08) | 0.02 (0.00 to 0.13) |

*BARC*, bleeding academic research consortium; *CI*, confidence interval; *CV*, cardiovascular; *OR*, odds ratio; *RCT*, randomized controlled trial; *TIMI*, thrombolysis in myocardial infarction;

**Supplemental table 10.** Overall analyses of the correlation of treatment effects and coefficient of determination of bleeding for all-cause and cardiovascular mortality among patients with acute coronary syndrome (OR)

| Bleeding criteria | Analysis | Comparison pairs | RCTs | Patient number | Regression formula | Slope (95%CI) | R^2^ (95%CI) |
| --- | --- | --- | --- | --- | --- | --- | --- |
| Major or minor bleeding | | | | | | | |
| Study defined major or minor | All-cause mortality | 32 | 28 | 107,395 | -0.11+0.13*log(OR_bleeding) | 0.13 (-0.02 to 0.27) | 0.09 (0.00 to 0.30) |
| BARC 2,3,5 | All-cause mortality | 14 | 14 | 68,403 | -0.10+0.17*log(OR_bleeding) | 0.17 (-0.08 to 0.43) | 0.16 (0.00 to 0.55) |
| TIMI major or minor | All-cause mortality | 17 | 16 | 70,511 | -0.16+0.24*log(OR_bleeding) | 0.24 (0.06 to 0.42) | 0.35 (0.00 to 0.75) |
| Study defined major or minor | CV mortality | 30 | 26 | 94,339 | -0.18+0.19*log(OR_bleeding) | 0.19 (-0.01 to 0.38) | 0.12 (0.00 to 0.35) |
| BARC 2,3,5 | CV mortality | 13 | 13 | 28,424 | -0.19+0.34*log(OR_bleeding) | 0.34 (-0.17 to 0.85) | 0.16 (0.00 to 0.58) |
| TIMI major or minor | CV mortality | 16 | 15 | 68,910 | -0.16+0.18*log(OR_bleeding) | 0.18 (-0.08 to 0.45) | 0.13 (0.00 to 0.48) |
| Major bleeding | | | | | | | |
| Study defined Major | All-cause mortality | 38 | 33 | 135,107 | -0.15+0.04*log(OR_bleeding) | 0.04 (-0.06 to 0.15) | 0.02 (0.00 to 0.11) |
| BARC 3,5 | All-cause mortality | 15 | 15 | 44,915 | -0.03+0.07*log(OR_bleeding) | 0.07 (-0.24 to 0.38) | 0.02 (0.00 to 0.17) |
| TIMI Major | All-cause mortality | 28 | 23 | 105,596 | -0.16+0.00*log(OR_bleeding) | 0.00 (-0.13 to 0.13) | 0.00 (0.00 to 0.01) |
| Study defined Major | CV mortality | 36 | 31 | 122,051 | -0.19+0.02*log(OR_bleeding) | 0.02 (-0.12 to 0.16) | 0.00 (0.00 to 0.04) |
| BARC 3,5 | CV mortality | 13 | 13 | 32,814 | -0.12-0.02*log(OR_bleeding) | -0.02 (-0.55 to 0.52) | 0.00 (0.00 to 0.03) |
| TIMI Major | CV mortality | 27 | 22 | 100,027 | -0.15-0.03*log(OR_bleeding) | -0.03 (-0.17 to 0.11) | 0.01 (0.00 to 0.07) |

*BARC*, bleeding academic research consortium; *CI*, confidence interval; *CV*, cardiovascular; *OR*, odds ratio; *RCT*, randomized controlled trial; *TIMI*, thrombolysis in myocardial infarction;

**Supplemental table 11**. All-cause mortality and major or minor bleeding sub-analysis among patients with acute coronary syndrome (OR)

| Analysis |  | Comparison pairs | RCTs | Patient number | Regression formula for all-cause mortality | Slope (95%CI) | R^2^ (95%CI) |
| --- | --- | --- | --- | --- | --- | --- | --- |
| Study defined major or minor bleeding | | | | | | | |
| Year of the first patient enrollment | Before 2010 | 16 | 12 | 63,562 | -0.20+0.25*log(OR_bleeding) | 0.25 (0.11 to 0.38) | 0.51 (0.13 to 0.89) |
|  | After 2011 | 16 | 16 | 43,833 | -0.03+0.15*log(OR_bleeding) | 0.15 (-0.24 to 0.54) | 0.05 (0.00 to 0.27) |
| Follow-up duration | ≤12 months | 22 | 18 | 61,842 | -0.14+0.05*log(OR_bleeding) | 0.05 (-0.23 to 0.32) | 0.01 (0.00 to 0.08) |
|  | >12 months | 10 | 10 | 45,553 | -0.13+0.19*log(OR_bleeding) | 0.19 (0.01 to 0.37) | 0.42 (0.00 to 0.97) |
| Location of the trial | East Asia | 9 | 9 | 20,119 | 0.02+0.07*log(OR_bleeding) | 0.07 (-0.53 to 0.67) | 0.01 (0.00 to 0.17) |
|  | Non-East Asia | 23 | 19 | 87,276 | -0.16+0.22*log(OR_bleeding) | 0.22 (0.08 to 0.36) | 0.34 (0.01 to 0.68) |
| BARC 2,3, or 5 bleeding | | | | | | | |
| Year of the first patient enrollment | Before 2010 | 2 | 2 | 5,041 | 0.10-0.31*log(OR_bleeding) | -0.31 (Not applicable) | 1.00 (1.00 to 1.00) |
|  | After 2011 | 12 | 12 | 34,838 | -0.03+0.34*log(OR_bleeding) | 0.34 (-0.02 to 0.71) | 0.31 (0.00 to 0.81) |
| Follow-up duration | ≤12 months | 9 | 9 | 26,016 | -0.05+0.26*log(OR_bleeding) | 0.26 (-0.19 to 0.70) | 0.21 (0.00 to 0.79) |
|  | >12 months | 5 | 5 | 13,863 | -0.15+0.14*log(OR_bleeding) | 0.14 (-0.46 to 0.74) | 0.16 (0.00 to 1.00) |
| Location of the trial | East Asia | 4 | 4 | 8,547 | 0.17+0.47*log(OR_bleeding) | 0.47 (-0.90 to 1.84) | 0.53 (0.00 to 1.00) |
|  | Non-East Asia | 10 | 10 | 31,332 | -0.13+0.19*log(OR_bleeding) | 0.19 (-0.12 to 0.50) | 0.20 (0.00 to 0.72) |
| TIMI major or minor bleeding | | | | | | | |
| Year of the first patient enrollment | Before 2010 | 11 | 10 | 57,012 | -0.28+0.24*log(OR_bleeding) | 0.24 (0.03 to 0.44) | 0.42 (0.00 to 0.94) |
|  | After 2011 | 6 | 6 | 13,499 | -0.09+0.40*log(OR_bleeding) | 0.40 (-0.30 to 1.11) | 0.39 (0.00 to 1.00) |
| Follow-up duration | ≤12 months | 11 | 10 | 37,356 | -0.18+0.16*log(OR_bleeding) | 0.16 (-0.33 to 0.66) | 0.06 (0.00 to 0.37) |
|  | >12 months | 6 | 6 | 33,155 | -0.13+0.21*log(OR_bleeding) | 0.21 (0.11 to 0.31) | 0.89 (0.67 to 1.00) |
| Location of the trial | East Asia | 4 | 4 | 7,435 | -0.06+0.55*log(OR_bleeding) | 0.55 (-0.05 to 1.15) | 0.89 (0.47 to 1.00) |
|  | Non-East Asia | 13 | 12 | 63,076 | -0.16+0.20*log(OR_bleeding) | 0.20 (-0.03 to 0.42) | 0.26 (0.00 to 0.72) |

*BARC*, bleeding academic research consortium; *CI*, confidence interval; *CV*, cardiovascular; *OR*, odds ratio; *RCT*, randomized controlled trial; *TIMI*, thrombolysis in myocardial infarction;

**Supplemental table 12**. All-cause mortality and major bleeding sub-analysis among patients with acute coronary syndrome (OR)

| Analysis |  | Comparison pairs | RCTs | Patient number | Regression formula for all-cause mortality | Slope (95%CI) | R^2^ (95%CI) |
| --- | --- | --- | --- | --- | --- | --- | --- |
| Study defined Major bleeding | | | | | | | |
| Year of the first patient enrollment | Before 2010 | 19 | 14 | 84,823 | -0.20+0.06*log(OR_bleeding) | 0.06 (-0.07 to 0.20) | 0.05 (0.00 to 0.27) |
|  | After 2011 | 19 | 19 | 50,284 | -0.01+0.24*log(OR_bleeding) | 0.24 (-0.05 to 0.54) | 0.15 (0.00 to 0.47) |
| Follow-up duration | ≤12 months | 24 | 20 | 67,090 | -0.10+0.13*log(OR_bleeding) | 0.13 (-0.19 to 0.46) | 0.03 (0.00 to 0.18) |
|  | >12 months | 14 | 13 | 68,017 | -0.18+0.06*log(OR_bleeding) | 0.06 (-0.08 to 0.20) | 0.06 (0.00 to 0.34) |
| Location of the trial | East Asia | 10 | 10 | 21,948 | -0.03-0.02*log(OR_bleeding) | -0.02 (-0.56 to 0.53) | 0.00 (0.00 to 0.03) |
|  | Non-East Asia | 28 | 23 | 113,159 | -0.18+0.08*log(OR_bleeding) | 0.08 (-0.04 to 0.19) | 0.07 (0.00 to 0.26) |
| BARC 3 or 5 bleeding | | | | | | | |
| Year of the first patient enrollment | Before 2010 | 1 | 1 | 1,465 | Not applicable | Not applicable | Not applicable |
|  | After 2011 | 14 | 14 | 43,450 | -0.03+0.08*log(OR_bleeding) | 0.08 (-0.25 to 0.41) | 0.02 (0.00 to 0.19) |
| Follow-up duration | ≤12 months | 11 | 11 | 34,295 | -0.01-0.09*log(OR_bleeding) | -0.09 (-0.55 to 0.37) | 0.02 (0.00 to 0.21) |
|  | >12 months | 4 | 4 | 10,620 | -0.18+0.16*log(OR_bleeding) | 0.16 (-0.30 to 0.61) | 0.53 (0.00 to 1.00) |
| Location of the trial | East Asia | 6 | 6 | 14,512 | -0.03-0.28*log(OR_bleeding) | -0.28 (-0.88 to 0.32) | 0.30 (0.00 to 1.00) |
|  | Non-East Asia | 9 | 9 | 30,403 | -0.01+0.39*log(OR_bleeding) | 0.39 (0.14 to 0.64) | 0.66 (0.21 to 1.00) |
| TIMI Major bleeding | | | | | | | |
| Year of the first patient enrollment | Before 2010 | 18 | 13 | 81,247 | -0.18+0.02*log(OR_bleeding) | 0.02 (-0.13 to 0.17) | 0.00 (0.00 to 0.06) |
|  | After 2011 | 10 | 10 | 24,349 | -0.03+0.21*log(OR_bleeding) | 0.21 (-0.43 to 0.85) | 0.07 (0.00 to 0.43) |
| Follow-up duration | ≤12 months | 17 | 13 | 45,696 | -0.19-0.25*log(OR_bleeding) | -0.25 (-0.55 to 0.05) | 0.17 (0.00 to 0.54) |
|  | >12 months | 11 | 10 | 59,900 | -0.20+0.08*log(OR_bleeding) | 0.08 (-0.12 to 0.28) | 0.08 (0.00 to 0.45) |
| Location of the trial | East Asia | 5 | 5 | 11,571 | 0.13+0.23*log(OR_bleeding) | 0.23 (-1.35 to 1.80) | 0.06 (0.00 to 0.72) |
|  | Non-East Asia | 23 | 18 | 94,025 | -0.19+0.04*log(OR_bleeding) | 0.04 (-0.09 to 0.17) | 0.02 (0.00 to 0.13) |

*BARC*, bleeding academic research consortium; *CI*, confidence interval; *CV*, cardiovascular; *OR*, odds ratio; *RCT*, randomized controlled trial; *TIMI*, thrombolysis in myocardial infarction;

**Supplemental table 13**. Cardiovascular mortality and major or minor bleeding sub-analysis among patients with acute coronary syndrome (OR)

| Analysis |  | Comparison pairs | RCTs | Patient number | Regression formula for CV mortality | Slope (95%CI) | R^2^ (95%CI) |
| --- | --- | --- | --- | --- | --- | --- | --- |
| Study defined major or minor bleeding | | | | | | | |
| Year of the first patient enrollment | Before 2010 | 15 | 11 | 61,961 | -0.19+0.12*log(OR_bleeding) | 0.12 (-0.08 to 0.31) | 0.12 (0.00 to 0.46) |
|  | After 2011 | 15 | 15 | 32,378 | -0.02+0.51*log(OR_bleeding) | 0.51 (0.01 to 1.01) | 0.27 (0.00 to 0.70) |
| Follow-up duration | ≤12 months | 22 | 18 | 55,001 | -0.14+0.31*log(OR_bleeding) | 0.31 (0.00 to 0.63) | 0.18 (0.00 to 0.49) |
|  | >12 months | 8 | 8 | 39,338 | -0.09-0.03*log(OR_bleeding) | -0.03 (-0.43 to 0.37) | 0.01 (0.00 to 0.14) |
| Location of the trial | East Asia | 9 | 9 | 20,119 | 0.02+0.54*log(OR_bleeding) | 0.54 (-0.27 to 1.35) | 0.27 (0.00 to 0.87) |
|  | Non-East Asia | 21 | 17 | 74,220 | -0.18+0.13*log(OR_bleeding) | 0.13 (-0.07 to 0.33) | 0.09 (0.00 to 0.34) |
| BARC 2,3, or 5 bleeding | | | | | | | |
| Year of the first patient enrollment | Before 2010 | 2 | 2 | 5,041 | 1.19-2.03*log(OR_bleeding) | -2.03 (Not applicable) | 1.00 (1.00 to 1.00) |
|  | After 2011 | 11 | 11 | 23,383 | -0.03+0.74*log(OR_bleeding) | 0.74 (0.10 to 1.38) | 0.43 (0.00 to 0.95) |
| Follow-up duration | ≤12 months | 9 | 9 | 19,175 | -0.09+0.75*log(OR_bleeding) | 0.75 (0.07 to 1.42) | 0.49 (0.00 to 1.00) |
|  | >12 months | 4 | 4 | 9,249 | -0.11-0.22*log(OR_bleeding) | -0.22 (-1.89 to 1.45) | 0.14 (0.00 to 1.00) |
| Location of the trial | East Asia | 4 | 4 | 8,547 | 0.27+1.27*log(OR_bleeding) | 1.27 (-2.23 to 4.77) | 0.55 (0.00 to 1.00) |
|  | Non-East Asia | 9 | 9 | 19,877 | -0.16+0.12*log(OR_bleeding) | 0.12 (-0.47 to 0.71) | 0.03 (0.00 to 0.30) |
| TIMI major or minor bleeding | | | | | | | |
| Year of the first patient enrollment | Before 2010 | 10 | 9 | 55,411 | -0.17+0.11*log(OR_bleeding) | 0.11 (-0.16 to 0.39) | 0.10 (0.00 to 0.53) |
|  | After 2011 | 6 | 6 | 13,499 | -0.05+0.78*log(OR_bleeding) | 0.78 (-0.15 to 1.71) | 0.58 (0.00 to 1.00) |
| Follow-up duration | ≤12 months | 11 | 10 | 37,356 | -0.15+0.40*log(OR_bleeding) | 0.40 (-0.28 to 1.08) | 0.16 (0.00 to 0.63) |
|  | >12 months | 5 | 5 | 31,554 | -0.12+0.07*log(OR_bleeding) | 0.07 (-0.10 to 0.24) | 0.38 (0.00 to 1.00) |
| Location of the trial | East Asia | 4 | 4 | 7,435 | 0.05+0.98*log(OR_bleeding) | 0.98 (-0.09 to 2.05) | 0.89 (0.47 to 1.00) |
|  | Non-East Asia | 12 | 11 | 61,475 | -0.14+0.05*log(OR_bleeding) | 0.05 (-0.20 to 0.31) | 0.02 (0.00 to 0.21) |

*BARC*, bleeding academic research consortium; *CI*, confidence interval; *CV*, cardiovascular; *OR*, odds ratio; *RCT*, randomized controlled trial; *TIMI*, thrombolysis in myocardial infarction;

**Supplemental table 14**. Cardiovascular mortality and major bleeding sub-analysis among patients with acute coronary syndrome (OR)

| Analysis |  | Comparison pairs | RCTs | Patient number | Regression of formula for CV mortality | Slope (95%CI) | R^2^ (95%CI) |
| --- | --- | --- | --- | --- | --- | --- | --- |
| Study defined Major bleeding | | | | | | | |
| Year of the first patient enrollment | Before 2010 | 18 | 13 | 83,222 | -0.17-0.02*log(OR_bleeding) | -0.02 (-0.16 to 0.12) | 0.01 (0.00 to 0.09) |
|  | After 2011 | 18 | 18 | 38,829 | -0.13+0.32*log(OR_bleeding) | 0.32 (-0.17 to 0.80) | 0.11 (0.00 to 0.40) |
| Follow-up duration | ≤12 months | 24 | 20 | 60,249 | -0.15+0.26*log(OR_bleeding) | 0.26 (-0.13 to 0.65) | 0.08 (0.00 to 0.30) |
|  | >12 months | 12 | 11 | 61,802 | -0.14-0.05*log(OR_bleeding) | -0.05 (-0.22 to 0.12) | 0.04 (0.00 to 0.28) |
| Location of the trial | East Asia | 10 | 10 | 21,948 | -0.19+0.08*log(OR_bleeding) | 0.08 (-0.76 to 0.93) | 0.01 (0.00 to 0.13) |
|  | Non-East Asia | 26 | 21 | 100,103 | -0.18+0.01*log(OR_bleeding) | 0.01 (-0.12 to 0.14) | 0.00 (0.00 to 0.02) |
| BARC 3 or 5 bleeding | | | | | | | |
| Year of the first patient enrollment | Before 2010 | 1 | 1 | 1,465 | Not applicable | Not applicable | Not applicable |
|  | After 2011 | 12 | 12 | 31,349 | -0.13-0.03*log(OR_bleeding) | -0.03 (-0.61 to 0.55) | 0.00 (0.00 to 0.05) |
| Follow-up duration | ≤12 months | 10 | 10 | 26,808 | -0.12-0.08*log(OR_bleeding) | -0.08 (-0.83 to 0.68) | 0.01 (0.00 to 0.13) |
|  | >12 months | 3 | 3 | 6,006 | -0.18+0.19*log(OR_bleeding) | 0.19 (-1.55 to 1.93) | 0.66 (0.00 to 1.00) |
| Location of the trial | East Asia | 6 | 6 | 14,512 | -0.26-0.15*log(OR_bleeding) | -0.15 (-1.39 to 1.10) | 0.03 (0.00 to 0.38) |
|  | Non-East Asia | 7 | 7 | 18,302 | -0.06+0.06*log(OR_bleeding) | 0.06 (-0.65 to 0.76) | 0.01 (0.00 to 0.19) |
| TIMI Major bleeding | | | | | | | |
| Year of the first patient enrollment | Before 2010 | 17 | 12 | 79,646 | -0.12-0.09*log(OR_bleeding) | -0.09 (-0.24 to 0.05) | 0.12 (0.00 to 0.43) |
|  | After 2011 | 10 | 10 | 20,381 | 0.05+0.58*log(OR_bleeding) | 0.58 (0.06 to 1.11) | 0.45 (0.00 to 1.00) |
| Follow-up duration | ≤12 months | 18 | 14 | 46,342 | -0.18-0.01*log(OR_bleeding) | -0.01 (-0.35 to 0.33) | 0.00 (0.00 to 0.02) |
|  | >12 months | 9 | 8 | 53,685 | -0.09-0.09*log(OR_bleeding) | -0.09 (-0.32 to 0.15) | 0.10 (0.00 to 0.55) |
| Location of the trial | East Asia | 5 | 5 | 11,571 | 0.24+0.79*log(OR_bleeding) | 0.79 (-0.29 to 1.86) | 0.64 (0.00 to 1.00) |
|  | Non-East Asia | 22 | 17 | 88,456 | -0.15-0.05*log(OR_bleeding) | -0.05 (-0.20 to 0.09) | 0.03 (0.00 to 0.17) |

*BARC*, bleeding academic research consortium; *CI*, confidence interval; *CV*, cardiovascular; *OR*, odds ratio; *RCT*, randomized controlled trial; *TIMI*, thrombolysis in myocardial infarction;

**eReferences**

**(1-52)**

1. Zheng YY, Wu TT, Yang Y et al. Personalized antiplatelet therapy guided by a novel detection of platelet aggregation function in stable coronary artery disease patients undergoing percutaneous coronary intervention: a randomized controlled clinical trial. Eur Heart J Cardiovasc Pharmacother 2020;6:211-221.

2. Wiviott SD, Braunwald E, McCabe CH et al. Prasugrel versus clopidogrel in patients with acute coronary syndromes. N Engl J Med 2007;357:2001-15.

3. Vranckx P, Valgimigli M, Juni P et al. Ticagrelor plus aspirin for 1 month, followed by ticagrelor monotherapy for 23 months vs aspirin plus clopidogrel or ticagrelor for 12 months, followed by aspirin monotherapy for 12 months after implantation of a drug-eluting stent: a multicentre, open-label, randomised superiority trial. Lancet 2018;392:940-949.

4. Tomaniak M, Chichareon P, Onuma Y et al. Benefit and Risks of Aspirin in Addition to Ticagrelor in Acute Coronary Syndromes: A Post Hoc Analysis of the Randomized GLOBAL LEADERS Trial. JAMA Cardiol 2019;4:1092-1101.

5. Smits PC, Frigoli E, Tijssen J et al. Abbreviated Antiplatelet Therapy in Patients at High Bleeding Risk With or Without Oral Anticoagulant Therapy After Coronary Stenting: An Open-Label, Randomized, Controlled Trial. Circulation 2021;144:1196-1211.

6. Sibbing D, Aradi D, Jacobshagen C et al. Guided de-escalation of antiplatelet treatment in patients with acute coronary syndrome undergoing percutaneous coronary intervention (TROPICAL-ACS): a randomised, open-label, multicentre trial. Lancet 2017;390:1747-1757.

7. Schupke S, Neumann FJ, Menichelli M et al. Ticagrelor or Prasugrel in Patients with Acute Coronary Syndromes. N Engl J Med 2019;381:1524-1534.

8. Savonitto S, Ferri LA, Piatti L et al. Comparison of Reduced-Dose Prasugrel and Standard-Dose Clopidogrel in Elderly Patients With Acute Coronary Syndromes Undergoing Early Percutaneous Revascularization. Circulation 2018;137:2435-2445.

9. Saito S, Isshiki T, Kimura T et al. Efficacy and safety of adjusted-dose prasugrel compared with clopidogrel in Japanese patients with acute coronary syndrome: the PRASFIT-ACS study. Circ J 2014;78:1684-92.

10. Pereira NL, Farkouh ME, So D et al. Effect of Genotype-Guided Oral P2Y12 Inhibitor Selection vs Conventional Clopidogrel Therapy on Ischemic Outcomes After Percutaneous Coronary Intervention: The TAILOR-PCI Randomized Clinical Trial. JAMA 2020;324:761-771.

11. Notarangelo FM, Maglietta G, Bevilacqua P et al. Pharmacogenomic Approach to Selecting Antiplatelet Therapy in Patients With Acute Coronary Syndromes: The PHARMCLO Trial. J Am Coll Cardiol 2018;71:1869-1877.

12. Koo BK, Kang J, Park KW et al. Aspirin versus clopidogrel for chronic maintenance monotherapy after percutaneous coronary intervention (HOST-EXAM): an investigator-initiated, prospective, randomised, open-label, multicentre trial. Lancet 2021;397:2487-2496.

13. Kim HS, Kang J, Hwang D et al. Prasugrel-based de-escalation of dual antiplatelet therapy after percutaneous coronary intervention in patients with acute coronary syndrome (HOST-REDUCE-POLYTECH-ACS): an open-label, multicentre, non-inferiority randomised trial. Lancet 2020;396:1079-1089.

14. Kim CJ, Park MW, Kim MC et al. Unguided de-escalation from ticagrelor to clopidogrel in stabilised patients with acute myocardial infarction undergoing percutaneous coronary intervention (TALOS-AMI): an investigator-initiated, open-label, multicentre, non-inferiority, randomised trial. Lancet 2021;398:1305-1316.

15. Han Y, Xu B, Xu K et al. Six Versus 12 Months of Dual Antiplatelet Therapy After Implantation of Biodegradable Polymer Sirolimus-Eluting Stent: Randomized Substudy of the I-LOVE-IT 2 Trial. Circ Cardiovasc Interv 2016;9:e003145.

16. Hahn JY, Song YB, Oh JH et al. Effect of P2Y12 Inhibitor Monotherapy vs Dual Antiplatelet Therapy on Cardiovascular Events in Patients Undergoing Percutaneous Coronary Intervention: The SMART-CHOICE Randomized Clinical Trial. JAMA 2019;321:2428-2437.

17. Hahn JY, Song YB, Oh JH et al. 6-month versus 12-month or longer dual antiplatelet therapy after percutaneous coronary intervention in patients with acute coronary syndrome (SMART-DATE): a randomised, open-label, non-inferiority trial. Lancet 2018;391:1274-1284.

18. De Luca G, Damen SA, Camaro C et al. Final results of the randomised evaluation of short-term dual antiplatelet therapy in patients with acute coronary syndrome treated with a new-generation stent (REDUCE trial). EuroIntervention 2019;15:e990-e998.

19. Cuisset T, Deharo P, Quilici J et al. Benefit of switching dual antiplatelet therapy after acute coronary syndrome: the TOPIC (timing of platelet inhibition after acute coronary syndrome) randomized study. Eur Heart J 2017;38:3070-3078.

20. Costa F, Vranckx P, Leonardi S et al. Impact of clinical presentation on ischaemic and bleeding outcomes in patients receiving 6- or 24-month duration of dual-antiplatelet therapy after stent implantation: a pre-specified analysis from the PRODIGY (Prolonging Dual-Antiplatelet Treatment After Grading Stent-Induced Intimal Hyperplasia) trial. Eur Heart J 2015;36:1242-51.

21. Colombo A, Chieffo A, Frasheri A et al. Second-generation drug-eluting stent implantation followed by 6- versus 12-month dual antiplatelet therapy: the SECURITY randomized clinical trial. J Am Coll Cardiol 2014;64:2086-97.

22. Cayla G, Cuisset T, Silvain J et al. Platelet function monitoring to adjust antiplatelet therapy in elderly patients stented for an acute coronary syndrome (ANTARCTIC): an open-label, blinded-endpoint, randomised controlled superiority trial. Lancet 2016;388:2015-2022.

23. Baber U, Dangas G, Angiolillo DJ et al. Ticagrelor alone vs. ticagrelor plus aspirin following percutaneous coronary intervention in patients with non-ST-segment elevation acute coronary syndromes: TWILIGHT-ACS. Eur Heart J 2020;41:3533-3545.

24. Motovska Z, Hlinomaz O, Kala P et al. 1-Year Outcomes of Patients Undergoing Primary Angioplasty for Myocardial Infarction Treated With Prasugrel Versus Ticagrelor. J Am Coll Cardiol 2018;71:371-381.

25. Kedhi E, Fabris E, van der Ent M et al. Six months versus 12 months dual antiplatelet therapy after drug-eluting stent implantation in ST-elevation myocardial infarction (DAPT-STEMI): randomised, multicentre, non-inferiority trial. BMJ 2018;363:k3793.

26. Gwon HC, Hahn JY, Park KW et al. Six-month versus 12-month dual antiplatelet therapy after implantation of drug-eluting stents: the Efficacy of Xience/Promus Versus Cypher to Reduce Late Loss After Stenting (EXCELLENT) randomized, multicenter study. Circulation 2012;125:505-13.

27. Jang JY, Shin DH, Kim JS et al. Optimal duration of DAPT after second-generation drug-eluting stent in acute coronary syndrome. PLoS One 2018;13:e0207386.

28. Hong SJ, Shin DH, Kim JS et al. 6-Month Versus 12-Month Dual-Antiplatelet Therapy Following Long Everolimus-Eluting Stent Implantation: The IVUS-XPL Randomized Clinical Trial. JACC Cardiovasc Interv 2016;9:1438-46.

29. Kim BK, Hong MK, Shin DH et al. A new strategy for discontinuation of dual antiplatelet therapy: the RESET Trial (REal Safety and Efficacy of 3-month dual antiplatelet Therapy following Endeavor zotarolimus-eluting stent implantation). J Am Coll Cardiol 2012;60:1340-8.

30. Schulz-Schupke S, Byrne RA, Ten Berg JM et al. ISAR-SAFE: a randomized, double-blind, placebo-controlled trial of 6 vs. 12 months of clopidogrel therapy after drug-eluting stenting. Eur Heart J 2015;36:1252-63.

31. Lohaus R, Michel J, Mayer K et al. Six Versus Twelve Months Clopidogrel Therapy After Drug-Eluting Stenting in Patients With Acute Coronary Syndrome: An ISAR-SAFE Study Subgroup Analysis. Sci Rep 2016;6:33054.

32. Kim BK, Hong SJ, Cho YH et al. Effect of Ticagrelor Monotherapy vs Ticagrelor With Aspirin on Major Bleeding and Cardiovascular Events in Patients With Acute Coronary Syndrome: The TICO Randomized Clinical Trial. JAMA 2020;323:2407-2416.

33. Park SJ, Park DW, Kim YH et al. Duration of dual antiplatelet therapy after implantation of drug-eluting stents. N Engl J Med 2010;362:1374-82.

34. Lee CW, Ahn JM, Park DW et al. Optimal duration of dual antiplatelet therapy after drug-eluting stent implantation: a randomized, controlled trial. Circulation 2014;129:304-12.

35. Didier R, Morice MC, Barragan P et al. 6- Versus 24-Month Dual Antiplatelet Therapy After Implantation of Drug-Eluting Stents in Patients Nonresistant to Aspirin: Final Results of the ITALIC Trial (Is There a Life for DES After Discontinuation of Clopidogrel). JACC Cardiovasc Interv 2017;10:1202-1210.

36. Alexander JH, Lopes RD, James S et al. Apixaban with antiplatelet therapy after acute coronary syndrome. N Engl J Med 2011;365:699-708.

37. Mega JL, Braunwald E, Wiviott SD et al. Rivaroxaban in patients with a recent acute coronary syndrome. N Engl J Med 2012;366:9-19.

38. Claassens DMF, Vos GJA, Bergmeijer TO et al. A Genotype-Guided Strategy for Oral P2Y12 Inhibitors in Primary PCI. N Engl J Med 2019;381:1621-1631.

39. Wallentin L, Becker RC, Budaj A et al. Ticagrelor versus clopidogrel in patients with acute coronary syndromes. N Engl J Med 2009;361:1045-57.

40. Goto S, Huang CH, Park SJ, Emanuelsson H, Kimura T. Ticagrelor vs. clopidogrel in Japanese, Korean and Taiwanese patients with acute coronary syndrome -- randomized, double-blind, phase III PHILO study. Circ J 2015;79:2452-60.

41. Park DW, Kwon O, Jang JS et al. Clinically Significant Bleeding With Ticagrelor Versus Clopidogrel in Korean Patients With Acute Coronary Syndromes Intended for Invasive Management: A Randomized Clinical Trial. Circulation 2019;140:1865-1877.

42. Gimbel M, Qaderdan K, Willemsen L et al. Clopidogrel versus ticagrelor or prasugrel in patients aged 70 years or older with non-ST-elevation acute coronary syndrome (POPular AGE): the randomised, open-label, non-inferiority trial. Lancet 2020;395:1374-1381.

43. Roe MT, Armstrong PW, Fox KA et al. Prasugrel versus clopidogrel for acute coronary syndromes without revascularization. N Engl J Med 2012;367:1297-309.

44. Roe MT, Goodman SG, Ohman EM et al. Elderly patients with acute coronary syndromes managed without revascularization: insights into the safety of long-term dual antiplatelet therapy with reduced-dose prasugrel versus standard-dose clopidogrel. Circulation 2013;128:823-33.

45. Watanabe H, Morimoto T, Natsuaki M et al. Comparison of Clopidogrel Monotherapy After 1 to 2 Months of Dual Antiplatelet Therapy With 12 Months of Dual Antiplatelet Therapy in Patients With Acute Coronary Syndrome: The STOPDAPT-2 ACS Randomized Clinical Trial. JAMA Cardiol 2022;7:407-417.

46. Price MJ, Berger PB, Teirstein PS et al. Standard- vs high-dose clopidogrel based on platelet function testing after percutaneous coronary intervention: the GRAVITAS randomized trial. JAMA 2011;305:1097-105.

47. Yeh RW, Kereiakes DJ, Steg PG et al. Benefits and Risks of Extended Duration Dual Antiplatelet Therapy After PCI in Patients With and Without Acute Myocardial Infarction. J Am Coll Cardiol 2015;65:2211-21.

48. Helft G, Steg PG, Le Feuvre C et al. Stopping or continuing clopidogrel 12 months after drug-eluting stent placement: the OPTIDUAL randomized trial. Eur Heart J 2016;37:365-74.

49. Committee AS, Investigators, Alexander JH et al. Apixaban, an oral, direct, selective factor Xa inhibitor, in combination with antiplatelet therapy after acute coronary syndrome: results of the Apixaban for Prevention of Acute Ischemic and Safety Events (APPRAISE) trial. Circulation 2009;119:2877-85.

50. Oldgren J, Budaj A, Granger CB et al. Dabigatran vs. placebo in patients with acute coronary syndromes on dual antiplatelet therapy: a randomized, double-blind, phase II trial. Eur Heart J 2011;32:2781-9.

51. Feres F, Costa RA, Abizaid A et al. Three vs twelve months of dual antiplatelet therapy after zotarolimus-eluting stents: the OPTIMIZE randomized trial. JAMA 2013;310:2510-22.

52. Collet JP, Silvain J, Barthelemy O et al. Dual-antiplatelet treatment beyond 1 year after drug-eluting stent implantation (ARCTIC-Interruption): a randomised trial. Lancet 2014;384:1577-85.
